# A bioactive phlebovirus-like envelope protein in a hookworm endogenous virus

**DOI:** 10.1126/sciadv.abj6894

**Published:** 2022-05-11

**Authors:** Monique Merchant, Carlos P. Mata, Yangci Liu, Haoming Zhai, Anna V. Protasio, Yorgo Modis

**Affiliations:** 1 Molecular Immunity Unit, Department of Medicine, University of Cambridge, MRC Laboratory of Molecular Biology, Francis Crick Avenue, Cambridge, CB2 0QH, UK.; 2 Cambridge Institute of Therapeutic Immunology and Infectious Disease (CITIID), University of Cambridge School of Clinical Medicine, Cambridge CB2 0AW, UK.; 3 Department of Pathology, University of Cambridge, Tennis Court Road, Cambridge CB2 1QP, UK.; 4 Christ’s College, University of Cambridge, St Andrew’s Street, Cambridge, CB2 3BU, UK.

## Abstract

Endogenous viral elements (EVEs), accounting for 15% of our genome, serve as a genetic reservoir from which new genes can emerge. Nematode EVEs are particularly diverse and informative of virus evolution. We identify Atlas virus—an intact retrovirus-like EVE in the human hookworm *Ancylostoma ceylanicum*, with an envelope protein genetically related to G_N_-G_C_ glycoproteins from the family Phenuiviridae. A cryo-EM structure of Atlas G_C_ reveals a class II viral membrane fusion protein fold not previously seen in retroviruses. Atlas G_C_ has the structural hallmarks of an active fusogen. Atlas G_C_ trimers insert into membranes with endosomal lipid compositions and low pH. When expressed on the plasma membrane, Atlas G_C_ has cell-cell fusion activity. With its preserved biological activities, Atlas G_C_ has the potential to acquire a cellular function. Our work reveals structural plasticity in reverse-transcribing RNA viruses.

## Teaser

A retroelement with a retrovirus-like genome and a phlebovirus-like envelope protein provides insights on virus evolution.

## INTRODUCTION

Retroviruses and other reverse-transcribing RNA viruses can frequently integrate their genome, reverse-transcribed from RNA into DNA, into the host-cell genome. Viral genomes integrated into germline cells are inherited by future generations as endogenous viral elements (EVEs). Most EVEs are endogenous retroviruses (ERVs) and account for approximately 15% of the human genome, seven times more than protein-coding genes (*1*, *2*). EVEs and other transposons were initially viewed as parasitic DNA. It is now evident that they serve as a genetic reservoir, from which new genes and regulatory elements can emerge. Sequences of retroviral origin help control gene expression by serving as promoters, enhancers, and other regulatory elements (*3*, *4*). Genes coopted from EVEs have evolved to fulfill vital cellular functions (*1*). For example, syncytins, which drive cell-cell fusion of trophoblasts during placental development, are encoded by ERV envelope glycoprotein (*env*) genes (*3*, *5*). Another recent example is the Gag capsid protein encoded by the *Caenorhabditis elegans* Cer1 retrotransposon, which encapsidates small noncoding RNAs into nucleocapsids that can transfer RNAs conferring learned pathogen avoidance behavior from infected parents to naïve progeny (*6*).

The reduced mutation rate of host versus retrovirus genomes [10^−9^ versus 10^−3^ mutations per site per year (*7*)] means that EVEs are windows to ancestral retroviral sequences—evolutionary fossils preserved from the time of integration (*2*). Some EVE genes are expressed in human tissues and retain their biological activities, such as membrane fusion activity in the case of Env proteins (Envs) (*3*, *5*, *8*). Aberrant expression of Envs is associated with disease (*9*, *10*). With the biology of EVEs still largely uncharted, it is likely that many cellular functions of EVEs in health and disease remain undiscovered. Studying EVE genes with previously unidentified properties could therefore provide insights on the evolutionary history of reverse-transcribing RNA viruses and identify fundamental principles in host-virus coevolution.

Nematode EVEs are particularly diverse and informative of virus evolution. EVEs from the family Belpaoviridae (BEL/Pao) (*11*), related to retroviruses and widespread across metazoa, have revealing genetic features in nematodes (*12*). The presence in *C. elegans* EVEs of overlapping open reading frames, otherwise unique to complex vertebrate retroviruses, suggests retroviruses originated in early metazoa with a common ancestor resembling belpaoviruses (*13*). Furthermore, nematode endogenous belpaoviruses encode Envs that are genetically unrelated to retrovirus Envs (*14*). Instead of a class I viral membrane fusion protein with a core fold of three bundled α helices (*15*–*18*)—a defining feature of modern retroviruses—belpaovirus Envs have sequence similarity to G_C_ (G_2_) envelope glycoproteins from phleboviruses and bandaviruses (family Phenuiviridae) (*14*). G_C_ proteins are class II membrane fusion proteins, with a three-domain β strand architecture (*19*, *20*) also found in alphaviruses (*21*), flaviviruses (*22*, *23*), and Rubella virus (*24*) but structurally unrelated to class I fusion proteins. A series of conformational changes in class II fusion proteins, triggered by endosomal acidification, catalyzes fusion of the viral and endosomal membranes to deliver the viral genome into the cytosol (*25*–*27*). A hydrophobic fusion loop first inserts into the endosomal membrane. The proteins then form trimers and fold back on themselves, pulling the cell membrane (held by the fusion loop) and the viral membrane (held by a transmembrane anchor) together so they fuse (*20*).

Class II fusion proteins are not limited to viruses: They also drive cell-cell fusion events of fundamental importance, including syncytial epithelia formation in *C. elegans* and other nematodes (*28*–*31*), and gamete fusion in protozoa, plants, algae, and invertebrates (*32*–*37*). The identical topology and overall arrangement of the three domains of viral and eukaryotic class II fusion proteins, along with similarities in their membrane fusion mechanisms, makes it all but certain they evolved from a common ancestor (*32*). Although the evolutionary origin of the ancestral class II fusion protein remains unknown, the presence of class II fusion proteins in EVEs raises the provocative prospect that a gene transfer from a virus to a cell led to the advent of sexual reproduction (*32*, *38*).

Here, we identify a novel, intact endogenous belpaovirus in the human hookworm *Ancylostoma ceylanicum* (a parasitic nematode) with an Env more similar than any other eukaryotic sequence to phlebovirus G_C_ protein sequences. We expressed and purified the G_C_-homologous fragment from this EVE, henceforth Atlas virus. A cryo–electron microscopy (cryo-EM) structure of Atlas G_C_ reveals a class II viral fusion protein fold similar to phlebovirus G_C_ proteins and not seen in retroviruses, as predicted 20 years ago (*12*, *14*). We show that Atlas G_C_ has all the hallmarks of an active class II membrane fusion protein. It undergoes a monomer-to-trimer transition and inserts into lipid membranes with a specific lipid composition in response to a low pH trigger. Our work provides biochemical validation for the hypothesis that acquisition of a fusion protein from an infectious virus, as exemplified by Atlas virus, represents a general paradigm of how retrotransposons can become retroviruses (*14*) and how ancestral reverse-transcribing viruses may have originated (*11*). The preserved biological activities of Atlas G_C_, including membrane fusion activity, raise the question of whether these activities, and those of EVE gene products more broadly, have cellular functions or cause disease.

## RESULTS

### An intact EVE with a phlebovirus-like Env in the hookworm *A. ceylanicum*

**Fig. 1.**
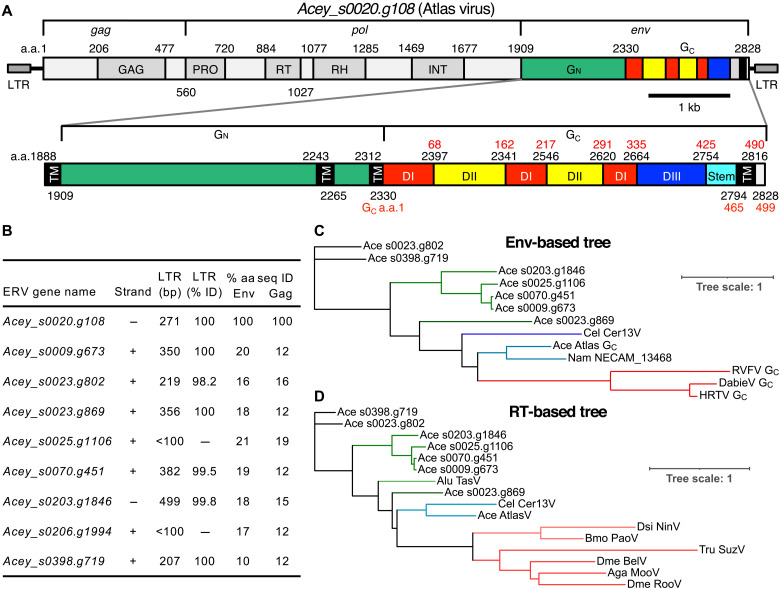
Atlas is an endogenous belpaovirus with a phlebovirus-like Env. (**A**) Gene architecture of the Atlas EVE, gene *Acey_s0020.g108* from the *A. ceylanicum* human hookworm parasite. Inset: Annotated close-up of the Atlas Env region encoding phlebovirus G_N_- and G_C_-like glycoproteins. Red residue numbers refer to the G_C_ sequence alone. a.a., amino acid. TM, transmembrane; DI, domain I; DII, domain II; DIII, domain III. (**B**) List of *A. ceylanicum* EVEs encoding complete Gag-Pol-Env polyproteins with phlebovirus-like Envs. LTR sequence identities for two of the EVEs could not be calculated: *Acey_s0206.g1994* had degenerate LTRs, and one LTR of *Acey_s0025.g1106* was truncated in the genome assembly. bp, base pair. (**C**) Phylogenetic tree of G_C_ proteins from *Phenuiviridae* and G_C_-like (G_2_-like) sequences from EVEs. Ace, *A. ceylanicum*; Cel, *C. elegans*; Nam, *Necator Americanus*, RVFV, Rift Valley fever virus; DabieV, Dabie bandavirus (SFTSV); HRTV, Heartland virus. (**D**) Phylogenetic classification of Atlas based on RT sequences. Alu TasV, *Ascaris lumbricoides* Tas virus; Dsi NinV, *Drosophila simulans* Ninja virus; Bmo PaoV, *Bombyx mori* Pao virus; Tru SuzV, *Takifugu rubripes* Suzu virus; Dme BelV, *D. melanogaster* Bel virus; Aga MooV, *Anopheles gambiae* Moose virus; RooV, Roo virus. Trees were drawn with iTOL v6.

The bioinformatic discovery of nematode EVEs with phlebovirus G_C_-like Env sequences not seen in retroviruses (*12*, *14*) requires biochemical validation. To identify phlebovirus-like EVE Envs suitable for biochemical analysis, we performed PSI-BLAST (position-specific iterative basic local alignment search tool) searches for protein sequences similar to biochemically characterized phlebovirus and bandavirus G_C_ proteins. A search with Rift Valley fever virus (RVFV) G_C_ as the query identified the gene *Acey_s0020.g108* (UniProt: A0A016UZK2) in the human hookworm *A. ceylanicum* as containing the most similar sequence outside infectious virus taxa [expected value (*E* value) of 10^−20^]. The homologous sequence lies within a 9204-nucleotide (nt) element having all the features of an intact EVE, including 100% identical 271-nt long terminal repeats (LTRs) and a coding sequence encoding a single 2828-residue Gag-Pol-Env polyprotein without any stop codons or introns (Fig. 1A and fig. S1A). We refer to this element as *A. ceylanicum* Atlas virus. It is one of nine *A. ceylanicum* EVEs that encode complete Gag-Pol-Env polyproteins (Fig. 1, B and C). These EVEs have the distinguishing genomic features of belpaoviruses from other nematode species, including an atypical Env and an aspartate to asparagine substitution (Y[X]DD → YVDN) in the most conserved reverse transcriptase (RT) motif, the polymerase site (fig. S1B) (*13*, *39*, *40*). Phylogenetic classification of the Atlas virus based on RT sequences confirms the Belpaoviridae phylogeny of Atlas virus, with Cer13 from *C. elegans* (*12*) as the closest neighbor in the RT tree (Fig. 1D). With a Gag sequence less than 20% identical to its closest homolog (*Acey_s0020.g1106*), the Atlas EVE is a candidate for classification as a member of the family Belpaoviridae (which contains a single genus, *Semotivirus*) (fig. S1C) (*41*).

The phlebovirus G_C_-like sequence spans the last 498 residues of the Atlas virus polyprotein (residues 2330 to 2828). It contains a single predicted C-terminal transmembrane helix, like phlebovirus G_C_ proteins. Phleboviruses and other Phenuiviridae family members express a glycoprotein precursor that is cleaved by cellular proteases into two envelope glycoproteins, G_N_ and G_C_ (or G_1_ and G_2_). G_N_ is the receptor-binding protein required for cellular attachment, and G_C_ is the membrane fusion protein required for cell entry. G_N_ is highly antigenic and more variable in sequence than G_C_. Our analysis of the Atlas virus Env sequence detected a slight but statistically significant similarity in the 421 residues preceding the G_C_-like sequence (residues 1909 to 2329) to G_N_ glycoproteins from Phenuiviridae family viruses (*E* value of ≥10^−7^). Moreover, the distribution of predicted transmembrane helices and proteolytic cleavage sites in and adjacent to the Atlas G_N_- and G_C_-like sequences is the same as in phlebovirus and bandavirus glycoproteins (Fig. 1A). Together, these sequence features suggest that the Atlas virus Env contains tandem phlebovirus-like G_N_ and G_C_ glycoproteins instead of a retrovirus-like glycoprotein. With all the features of a recently active EVE and an apparently intact set of phlebovirus-like glycoproteins, Atlas virus is an excellent candidate for biochemical analysis.

### Atlas G_C_ has a class II membrane fusion protein fold not seen in retroviruses

**Fig. 2.**
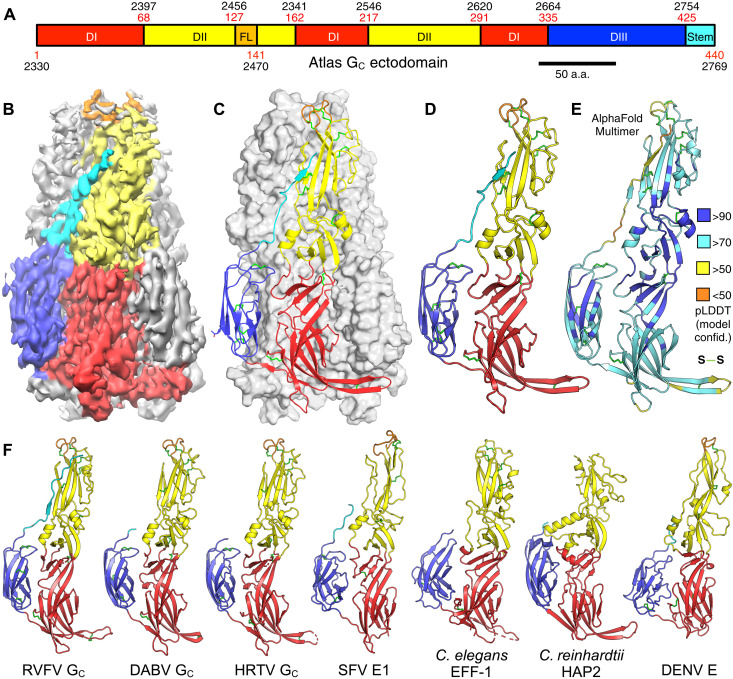
Cryo-EM structure of a phlebovirus G_C_-like Env fragment from Atlas virus. (**A**) Domain organization of Atlas G_C_. (**B**) Cryo-EM image reconstruction of a soluble ectodomain fragment of Atlas G_C_ at 3.76 Å overall resolution. The purified protein was a homotrimer (fig. S2), and threefold (C3) symmetry was imposed. The map is colored by domain as in (A). A representative cryo-EM micrograph is shown in fig. S4A. (**C**) Overview of the refined atomic model of the Atlas G_C_ trimer. Disulfide bonds (green) and an N-linked glycan (blue) are shown as sticks. (**D**) A single Atlas G_C_ protomer. (**E**) Protomer from the AlphaFold-Multimer structure prediction for the G_C_ trimer. The backbone is colored by model confidence [measured with the predicted local distance difference test (pLDDT)]; side chains of disulfide-bonded cysteines are in green. (**F**) The structures most similar to Atlas G_C_ based on structure comparison with DALI (*71*), G_C_ glycoproteins from RVFV [root mean square deviation (RMSD), 2.6 Å; *z* score, 29; PDB: 6EGU (*27*)], DABV [formerly SFTS phlebovirus; RMSD, 2.6 Å; *z* score, 28; PDB: 5G47 (*43*)], and HRTV (RMSD, 2.6 Å; *z* score, 28; PDB: 5YOW (*44*)]. Other representative class II fusion proteins are shown for comparison: SFV E1 [*z* score, 19; PDB: 1RER (*26*)], *C. elegans* EFF-1 [*z* score, 19; PDB: 4OJD (*28*)], *Chlamydomonas reinhardtii* HAP2 [*z* score, 16; PDB: 5MF1 (*32*)], and dengue virus (DENV) E [*z* score, 14; PDB: 3G7T (*46*)].

As the molecular structure of phlebovirus G_C_ proteins and how they drive fusion of the viral and cellular membranes are well established from previous studies (*19*, *27*), we focused our biochemical analyses on the G_C_-like sequence from the Atlas virus. A recombinant ectodomain fragment of Atlas G_C_ (polyprotein residues 2330 to 2772) was expressed in *Drosophila melanogaster* D.mel-2 cells as a secreted protein. The purified protein was a soluble, folded homotrimer (fig. S2). The structure of the G_C_ trimer was determined by single-particle cryo-EM image reconstruction at an overall resolution of 3.76 Å (Fig. 2, A and B, table S1, and fig. S3). The map was sufficiently detailed for an atomic model to be built and refined for Atlas G_C_ residues 2330 to 2769 using the crystal structure of RVFV G_C_ (*27*) as a starting model (see Materials and Methods; fig. S4). The structure reveals a three-domain class II membrane fusion protein fold (Fig. 2, C and D). An atomic model of the G_C_ trimer independently generated with AlphaFold-Multimer (*42*) was similar [root mean square deviation (RMSD) (Cα) = 1.17 Å; Fig. 2E]. All previously described retroviral Env structures have a helical coiled-coil class I fusion protein fold (*15*–*18*). The structure of the Atlas G_C_ ectodomain fragment is similar to phlebovirus and bandavirus G_C_ structures, specifically the trimeric postfusion G_C_ structures from RVFV (*27*), Dabie bandavirus [DABV, formerly SFTS (severe fever with thrombocytopenia syndrome) phlebovirus] (*43*) and Heartland virus (HRTV) (Fig. 2, D and F) (*44*). Domain I, a 10-stranded β barrel augmented by a three-stranded sheet, organizes the structure. Two insertions in domain I form the elongated, mostly β-stranded domain II. Domain III has the seven-stranded β-sandwich topology of fibronectin type III (FN3) domains also found in macroglobulin domains (*34*, *45*), but the hydrophobic core and disulfide bonding pattern of domain III differ from these and other annotated domains from nonviral species. A 16–amino acid portion of the stem region, which links domain III to the C-terminal transmembrane anchor in class II fusion proteins, could be modeled, spanning 5 nm from the end of domain III to within approximately 1 nm of the tip of domain II. The stem forms trimer contacts, adding a β strand to domain II of a different subunit, as seen in RVFV G_C_ (*27*). The overall configuration bears strong similarity to other viral and cellular class II fusion proteins including, in order of decreasing similarity: alphavirus E1 proteins (*21*), EFF-1/AFF-1 cell-cell fusion proteins from *C. elegans* and other animals (*28*, *29*), HAP2 (hapless 2, also known as generative cell-specific protein1 or GCS1) gamete fusion proteins from protozoa (*32*–*34*) and plants (*33*, *35*), and flavivirus E proteins (*22*, *23*, *46*) (Fig. 2F). Despite these structural similarities, the only proteins or domains of known structure with detectable amino acid sequence similarity to Atlas G_C_ (*E* value of <1 in PSI-BLAST) are the phlebovirus and bandavirus G_C_ proteins (22 to 24% sequence identity; fig. S5).

Atlas G_C_ has the same structural features that distinguish phlebovirus and bandavirus glycoproteins from other class II fusion proteins: a larger number of disulfide bonds, 10 of which are conserved in phleboviruses and bandaviruses but not in other class II proteins; N-linked glycosylation in domain III; and a more extensive and rigid interface between domains I and II (Fig. 2, D and F, and fig. S5). The most notable differences between Atlas virus and phlebovirus G_C_ structures are differences in the disulfide bonding pattern and in the composition of side chains lining the glycerophospholipid (GPL) headgroup binding pocket conserved in arboviral class II fusion proteins (*27*). We discuss these differences and their potential functional implications below. Atlas G_C_ also has a different glycosylation pattern, with a single predicted N-linked glycosylation site at Asn^414^ in domain III with a weak corresponding feature in the density map (fig. S6). In contrast, phlebovirus and bandavirus G_C_ proteins contain two N-linked glycans in domain III, at two different sites. One of these, Asn^1035^ in RVFV, covers the fusion loop in the prefusion conformation of RVFV G_C_ and stabilizes the prefusion dimer by forming contacts across the dimer interface (*19*), as also seen in flavivirus E proteins (*22*, *23*). This glycosylation site is conserved in DABV and HRTV G_C_ but absent in Atlas G_C_. Despite these minor differences, the notable overall structural similarity of Atlas G_C_ to phlebovirus and bandavirus G_C_ proteins in the postfusion conformation experimentally validates the evolutionary link between nematode EVEs from the family Belpaoviridae and the fusion proteins of phleboviruses postulated on the basis of previous genomic analyses (*12*, *14*).

### Structure of the putative lipid membrane anchor of Atlas G_C_

**Fig. 3.**
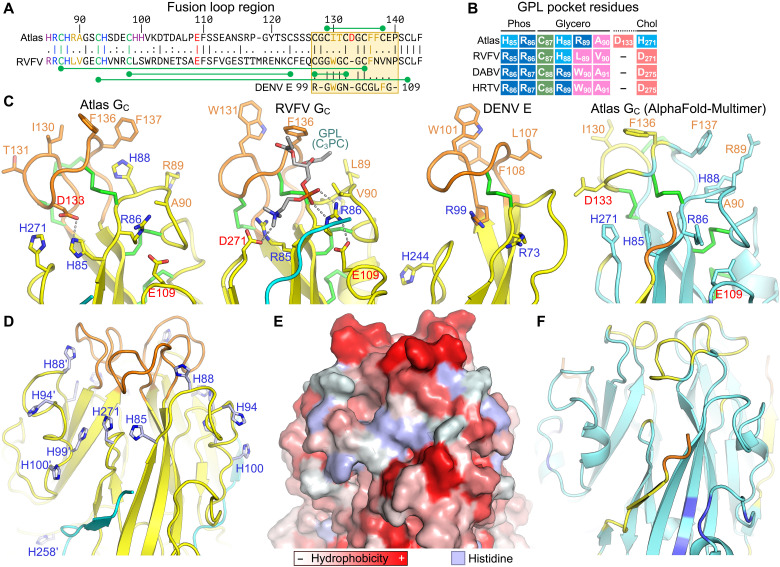
Structure of the putative fusion loop and lipid binding pocket of Atlas G_C_. (**A**) Local sequence alignment of the fusion loops (orange boxes) of Atlas G_C_, RVFV G_C_, and DENV E. Green dumbbells indicate disulfides. Disulfides shown below the RVFV sequence are conserved in Atlas and RVFV. (**B**) Residues contributing to binding of the phosphate (Phos), glycerol, and choline (Chol) moieties of glycerophospholipid (GPL) molecules by RVFV G_C_ (*27*). Colors indicate side chain properties: blue, positive charge; pink, negative charge; magenta, hydrophobic; green, sulfhydryl; light blue, positive charge at endosomal pH. (**C**) Close-up of the fusion loop and GPL binding pockets of Atlas G_C_, RVFV G_C_, and DENV E. A single protomer from the postfusion trimer is shown, with key residues shown in stick representation. Label colors indicate side chain properties: blue, positive charge; red, negative charge; orange, hydrophobic. The AlphaFold-Multimer model is colored by model confidence as in Fig. 2. (**D**) Close-up of the fusion loop and GPL binding pocket of the Atlas G_C_ trimer showing histidine residues in or near the pocket. Prime symbols following residue numbers denote the protomer to which the residue belongs. (**E**) Surface representation of the same view as in (D), colored by side chain hydrophobicity, except for histidine residues shown in light blue. (**F**) Close-up of the fusion loop and GPL binding pocket of the Atlas G_C_ AlphaFold-Multimer trimer model, same view as in (D).

Viral fusion proteins insert a membrane anchor—the fusion loop, in class II proteins—into the host cell membrane to initiate virus-cell membrane fusion. The putative fusion loop of Atlas G_C_ can be identified by analogy to RVFV G_C_ as spanning residues 127 to 140. The local resolution of the cryo-EM density for this region is lower than for the rest of the map, but the large number of structural constraints imposed by the positions of disulfide-bonded cysteines and other residues conserved in phleboviruses G_C_ proteins allowed an atomic model to be built unambiguously (Fig. 3A and fig. S6, B and C). Specifically, the fusion loop is constrained by four disulfide bonds conserved in phleboviruses and bandaviruses, a fifth disulfide specific to Atlas G_C_ (Cys^129^ to Cys^138^), a phenylalanine (Phe^136^) required in phleboviruses and bandaviruses at the apex of the fusion loop for membrane binding and fusion (*27*, *43*, *47*), and two conserved glycines (Gly^128^ and Gly^134^) that provide the torsional flexibility necessary for the fusion loop’s tightly folded conformation (Fig. 3, A to C). The fusion loop in the AlphaFold-Multimer model has the same fold and disulfide connectivity, validating the cryo-EM model (Fig. 3, C and F).

The structure and chemical properties of the Atlas G_C_ fusion loop resemble phlebovirus and bandavirus fusion loops. Ile^130^, Phe^136^, Phe^137^, and the Atlas-specific disulfide (Cys^129^ to Cys^138^) form a hydrophobic surface similar to phlebovirus, bandavirus, and flavivirus fusion proteins (Fig. 3C) (*25*, *27*). The area of this surface is greater than in most viral class II fusion proteins. By analogy with other viral class II fusion proteins, the location and extent of the hydrophobic surface formed by the Atlas G_C_ fusion loop suggest that it could function as a membrane anchor.

In addition to inserting nonpolar side chains into the hydrophobic region of the membrane, viral class II fusion proteins form polar contacts with lipid headgroups via the fusion loop and an adjacent GPL binding pocket (*27*). By selecting for headgroups with complementary electrostatic potential, polar contacts confer a degree of specificity to lipid binding. In phleboviruses, a set of conserved polar residues in the GPL binding pocket bind selectively to zwitterionic GPLs (*27*). The Atlas G_C_ structure reveals a putative GPL binding pocket with both conserved and novel features (Fig. 3). The arginine that forms bidentate hydrogen bonds with the GPL phosphate moiety in phleboviruses (*27*) is conserved in Atlas virus (Arg^86^). The disulfide bond and short-chain hydrophobic residue that bind the GPL glycerol moiety are also conserved (Cys^87^ to Cys^135^ and Ala^90^). However, an aspartate-arginine pair that binds choline and ethanolamine GPL moieties in phleboviruses is replaced in Atlas G_C_ by two histidines (His^271^ and His^85^). Moreover, Atlas virus has an extra residue in the fusion loop, Asp^133^, compared to phlebovirus G_C_ proteins. The Asp^133^ side chain points into the putative GPL binding pocket and is located near the position of the choline GPL moiety in the superimposed structure of RVFV G_C_ bound to a phosphatidylcholine (PC) ligand (*27*), suggesting that As^p133^ could compensate for the lack of a conserved aspartate at position 271 (Fig. 3, A to C). Hence, the putative GPL binding pocket of Atlas G_C_ appears to have the necessary physicochemical attributes to support GPL binding, with Arg^86^ binding the phosphate moiety, Cys^87^/Cys^135^/Ala^90^ binding the glycerol moiety, and His^85^/Asp^133^/His^271^ coordinating the end of the headgroup. We noted the presence in the cryo-EM reconstruction of a bulge in the density around the GPL binding pocket that is unaccounted for by the atomic model (fig. S6C). In addition, the absorbance at 260 nm of purified Atlas G_C_ was higher than expected despite treatment with nucleases during purification (fig. S2A). These two observations would be consistent with lipid molecules with unsaturated acyl chains copurifying with Atlas G_C_, but the local resolution of the map was insufficient to ascertain whether the GPL binding pocket contained a ligand.

### Atlas G_C_ binds membranes with endosome-like lipid composition at low pH

**Fig. 4.**
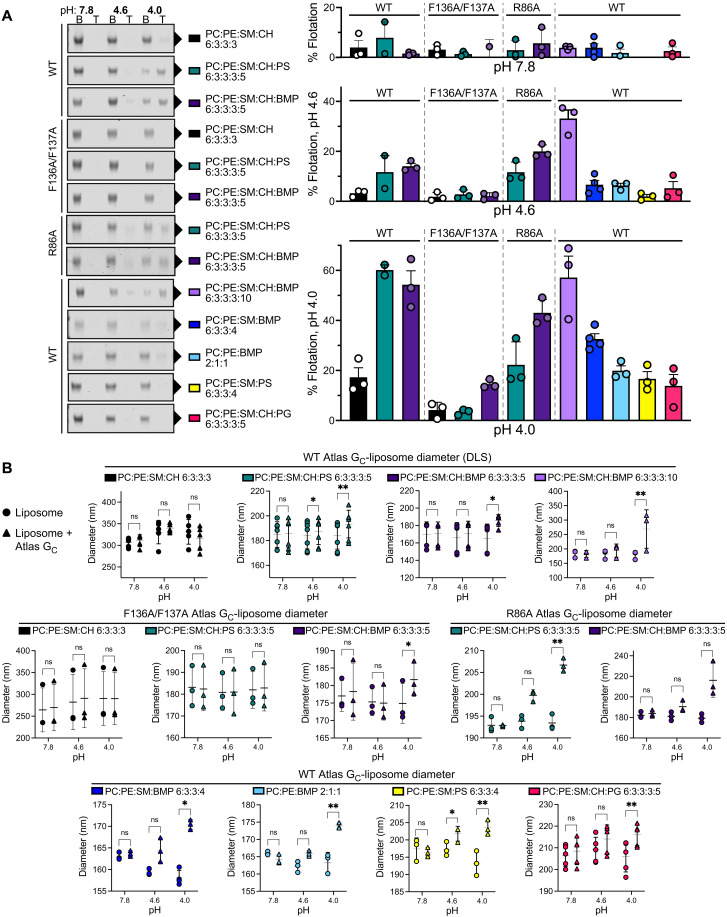
Atlas G_C_ ectodomain binds liposomes in a lipid- and pH-dependent manner. (**A**) Liposome coflotation lipid binding assay. A liposome-G_C_ ectodomain mixture in 40% OptiPrep density gradient medium was overlaid with a 30% OptiPrep cushion and centrifuged at 100,000*g*. Flotation was defined as the amount of G_C_ ectodomain cofloating with liposomes in the top-half (T) fraction divided by the total amount of G_C_ ectodomain in the top- and bottom-half (B) fractions. G_C_ ectodomain was quantified by Coomassie-stained SDS-PAGE. Error bars show the SD of three replicates except wild-type (WT) + PC:PE:SM:CH:PS (two replicates) and WT + PC:PE:SM:BMP (four replicates). See fig. S8 for uncropped gels for all replicates. (**B**) Binding of G_C_ ectodomain (WT, F136A/F137A or R86A) to liposomes measured by DLS as differences in liposome diameter in the presence and absence of Atlas G_C_ ectodomain. Error bars show the SD of three to seven replicates. Significance was determined by two-way ANOVA of the mean change in liposome diameter, using Sidak’s multiple comparisons test with a 95% confidence interval in GraphPad Prism 8 (see fig. S7). *P < 0.05; **P < 0.01; ns, not significant. See dataset S1 for source data.

A key step in viral membrane fusion is binding of the fusion protein to the host cell membrane. We assessed binding of Atlas G_C_ ectodomain to liposomes in density gradient centrifugation and dynamic light scattering (DLS) experiments. Viruses containing class II fusion proteins, like many retroviruses, undergo membrane insertion and fusion in endosomal compartments where the pH is acidic (*48*–*51*). We therefore assayed liposome binding at a range of pH values. In contrast to RVFV G_C_ ectodomain, Atlas G_C_ ectodomain did not bind liposomes containing PC, phosphatidylethanolamine (PE), cholesterol, and sphingomyelin (SM) at neutral or acidic pH (pH 4 to 8; Fig. 4, A and B). At neutral pH (pH 7.8), Atlas G_C_ ectodomain also failed to bind liposomes containing anionic lipids enriched in early or late endosomes: phosphatidylserine (PS) or bis(monoacylglycerol)phosphate (BMP, also known as lysobisphosphatidic acid), respectively. At pH 4, however, Atlas G_C_ ectodomain bound tightly to liposomes containing PS or BMP, with weaker binding observed at pH 4.6 (Fig. 4, A and B, and fig. S7). Atlas G_C_ ectodomain bound only weakly to liposomes containing phosphatidylglycerol (PG) instead of BMP although PG and BMP are regioisomers with identical chemical composition and electrostatic charge (of −1). PG and BMP differ only in the position of the second acyl-glycerol linkage, resulting in a linear configuration for BMP instead of the usual branched configuration for PG. Our liposome binding data show that Atlas G_C_ binds to membranes containing specific GPLs that are enriched in the endosomal pathway in a pH-dependent manner. No other class II fusion protein has been reported to require low pH, PS, or BMP for membrane insertion. However, phleboviruses require only PE or PC and cholesterol for membrane insertion (*27*), and bandaviruses require BMP and low pH for fusion (*52*). Similarly, flaviviruses require BMP, PS, or other anionic lipids and low pH for efficient fusion (*48*, *50*, *53*).

We note that the liposome diameters measured by DLS were smaller at pH 4 than at pH 7.8 and differences in lipid composition correlated with further differences in liposome diameter. Reduction of the pH below 6 reduces the lipid packing density within bilayers containing anionic lipids, which can, in turn, reduce the diameter of lipid vesicles (or alter their shape), an effect ascribed to headgroup protonation leading to reduced electrostatic repulsion (*54*). Differences in the cholesterol content of the bilayer can also contribute to fluctuations in liposome size, as cholesterol affects lipid packing and raft formation. Hence, we only compared the liposome diameters from DLS experiments performed at the same pH and with the same lipid composition, where the only difference was the presence or absence of Atlas G_C_ (Fig. 4B and fig. S7).

The optimal pH for membrane binding of Atlas G_C_ (pH 4 to 4.5) is similar to the optimal pH of hemifusion of Uukuniemi virus (*52*), a model phenuivirus (uukuvirus genus), and would be consistent with membrane insertion in late endosomes, as is the case for phenuiviruses (*49*). The side chains of His^85^ and His^271^, in the GPL binding pocket of Atlas G_C_, would be fully protonated at pH 4 to 4.5. The resulting net positive charge of the His^85^/Asp^133^/His^271^ triad (+1/−1/+1), analogous to the Arg/Asp pair that coordinates the end of GPL headgroups in phleboviruses and bandaviruses (Fig. 3B), mirrors the charge of the phosphoserine headgroup of PS (−1/+1/−1). Moreover, Atlas G_C_ contains four additional solvent-exposed histidines (residues 88, 94, 99, and 100) in the vicinity of the GPL binding pocket (Fig. 3, D and E). Protonation of these histidines at low pH may promote further interactions with anionic lipid headgroups. The presence of six histidines in and around the GPL binding pocket provides a possible explanation for the observed pH-dependent insertion of Atlas G_C_ into membranes containing PS and BMP. Consistent with a conserved role for the GPL binding pocket in determining lipid specificity of class II fusion proteins, mutations in alphaviruses at a position equivalent to His^271^, in the *ij* loop, determine the extent to which alphaviruses depend on cholesterol for membrane binding (*55*–*57*).

### Atlas G_C_ membrane binding does not strictly require cholesterol and occurs via the fusion loop

In addition to GPLs, phenuiviruses and alphaviruses (but not flaviviruses) require cholesterol for efficient membrane binding and subsequent fusion (*27*, *52*, *55*). Alphaviruses additionally require sphingolipids (such as SM) for efficient fusion (*58*, *59*). We found that neither cholesterol nor SM were required for Atlas G_C_ to bind liposomes (Fig. 4, A and B). Removal of cholesterol reduced the fraction of G_C_ bound by 50% in the liposome flotation assay (Fig. 4A), although binding was still detected in the DLS assay (Fig. 4B). Hence, although cholesterol and SM are not strictly required for binding, they enhance binding, possibly by increasing membrane fluidity. Notably, the concentration of cholesterol in nematode cell membranes is approximately 20 times lower than in vertebrates (*60*, *61*). This is insufficient for cholesterol to regulate the structure or fluidity of nematode membranes, in which cholesterol is thought to be instead a precursor for low-abundance metabolites (*60*–*62*). Likewise, *Drosophila* can grow indefinitely with only trace amounts of exogenous sterols (*60*), suggesting that arthropods, which are obligate vectors of the vast majority of viruses containing class II fusion proteins, rely on lipids other than cholesterol to regulate membrane fluidity.

To determine whether Atlas G_C_ binds membranes in a manner analogous to other viral class II fusion proteins—via the fusion loop and GPL binding pocket—we purified Atlas G_C_ ectodomain variants with mutations in the two phenylalanine residues in the fusion loop (F136A/F137A) or in the arginine predicted to bind the GPL phosphate moiety (R86A). The F136A/F137A mutant failed to bind liposomes containing PS and coflotation with liposomes containing BMP at pH 4 and pH 4.6 was reduced to approximately one-third of wild type (Fig. 4C). The R86A mutation reduced binding to liposomes containing PS or BMP at pH 4 to one-third and 75% of wild type, respectively (Fig. 4D). For both variants, preparations contained trimers as the major species, but a small monomeric fraction was also present (fig. S2), suggesting that the mutated lipid binding residues are required for efficient trimer assembly. We conclude that Atlas G_C_ binds to lipid membranes through insertion of hydrophobic fusion loop residues and coordination of lipid headgroups in the GPL binding pocket, as in other viral class II fusion proteins.

### Evidence for monomeric and trimeric states of Atlas G_C_

**Fig. 5.**
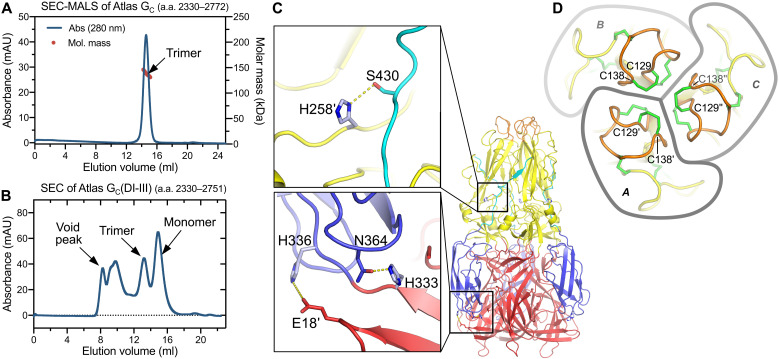
Atlas G_C_ oligomerization and disulfide bonding. (**A**) SEC–multiangle light scattering (MALS) of Atlas G_C_ ectodomain (residues 2330 to 2772). The protein formed trimers. (**B**) SEC of Atlas G_C_(DI-III) (residues 2330 to 2751) expressed in a mixture of oligomeric states, including monomers, trimers, and larger aggregates (void peak). G_C_(DI-III) monomers were unstable. (**C**) His^258^, His^333^, and His^336^ form interprotomer or interdomain polar contacts predicted to stabilize the G_C_ trimer specifically at pH < 6, when the histidine side chains are charged. Residues from different protomers are denoted with a prime symbol. (**D**) View along the threefold axis of the G_C_ trimer with intramolecular disulfides between the cysteines most proximal to the axis, Cys^129^ and Cys^138^.

Class II fusion protein ectodomains can be monomeric or dimeric or form icosahedral shells in their prefusion conformation, but the fusogenic conformational change is always accompanied by reorganization into trimers (*20*). Fusion proteins from classes I and III, including retrovirus fusogens, remain trimeric throughout the fusion reaction, but no class II fusion proteins are known to be trimeric in their prefusion conformation. Having established that Atlas G_C_ can insert into membranes as a trimer with a postfusion-like conformation, we set out to determine whether it could undergo a conformational change as seen in the fusion reaction of class II proteins from infectious viruses. The Atlas G_C_ construct described above was expressed as a trimer with no trace of monomers or dimers (Fig. 5A and fig. S2A). However, we found that a construct with the stem region truncated, G_C_(DI-III), spanning residues 2330 to 2751, was expressed as a mixture of monomers, trimers, and higher-order oligomers (Fig. 5B and fig. S2B). In contrast to G_C_ trimers, which were stable at different protein concentrations and pH values, G_C_(DI-III) monomers were unstable over time. As noted above, monomeric fractions were also present in preparations of the fusion loop mutant (F136A/F137A) and GPL binding pocket mutant (R86A) (fig. S2, C and D). Whether these monomeric species are in a prefusion conformation remains to be determined, but the presence of metastable monomers and stable trimers recapitulates a key property of class II fusogens from infectious viruses.

### pH-dependent stabilization of the Atlas G_C_ trimer by protonated histidine residues

The increase in positive charge resulting from histidine protonation is an important part of the pH sensing mechanism of viral class II fusion proteins. Protonation of conserved histidines at the domain I–domain III interface of alphavirus, flavivirus, and phlebovirus glycoproteins promotes the fusogenic conformational change by destabilizing the prefusion conformation and stabilizing the postfusion conformation (*19*, *27*, *46*, *63*–*65*). For example, histidines in domain III of phlebovirus G_C_ proteins form interprotomer salt bridges with negatively charged side chains in the postfusion trimer and mutation of one such histidine in RVFV, His^1087^, renders the virus uninfectious (*66*). Similarly, in Atlas G_C_, His^258^, His^333^, and His^336^ form interprotomer or interdomain polar contacts (Fig. 5C). These histidine-dependent contacts would stabilize the trimeric postfusion-like conformation of Atlas G_C_ specifically in acidic endosomal compartments. The parallels of how Atlas G_C_ and class II fusogens from infectious viruses respond at the ultrastructural level to environmental cues support the hypothesis that Atlas G_C_ would have membrane fusion activity in late endosomes, like phleboviruses and many retroviruses.

### Fifteen disulfide bonds stabilize Atlas G_C_ in its postfusion-like conformation

With 30 cysteine residues forming 15 disulfide bonds, Atlas G_C_ contains twice the average abundance of cysteines, more than has been found in any other class II protein. Twenty of these cysteines form disulfides that are structurally conserved in phleboviruses and bandaviruses (including RVFV, DABV, and HRTV) but not in other class II proteins (fig. S5). An 11th disulfide, in domain III, is conserved in Atlas virus, DABV and HRTV but not RVFV. However, Atlas G_C_ contains four additional disulfides: one in the fusion loop, two in domain II in the β-hairpin containing the *ij* loop (one of the cysteines forming these disulfides is conserved in phleboviruses but forms a disulfide with a cysteine in a different β strand in domain II), and one in domain III (Figs. 2C and 3A and fig. S5). The disulfide bonding patterns in the AlphaFold-Multimer and cryo-EM models were identical (Fig. 2, D and E). As discussed above, the Atlas-specific disulfide in the fusion loop (Cys^129^-Cys^138^) extends the hydrophobic surface formed by conserved residues in the fusion loop that are required for membrane insertion (Fig. 5D). We note that due to the location of Cys^129^ and Cys^138^ close to the threefold symmetry axis of the trimer, the side chains of the two residues can be rearranged by torsional rotation to form intermolecular disulfides across the trimer interface, thereby cross-linking all three protomers in the trimer.

### Atlas G_C_ retains membrane fusion activity

**Fig. 6.**
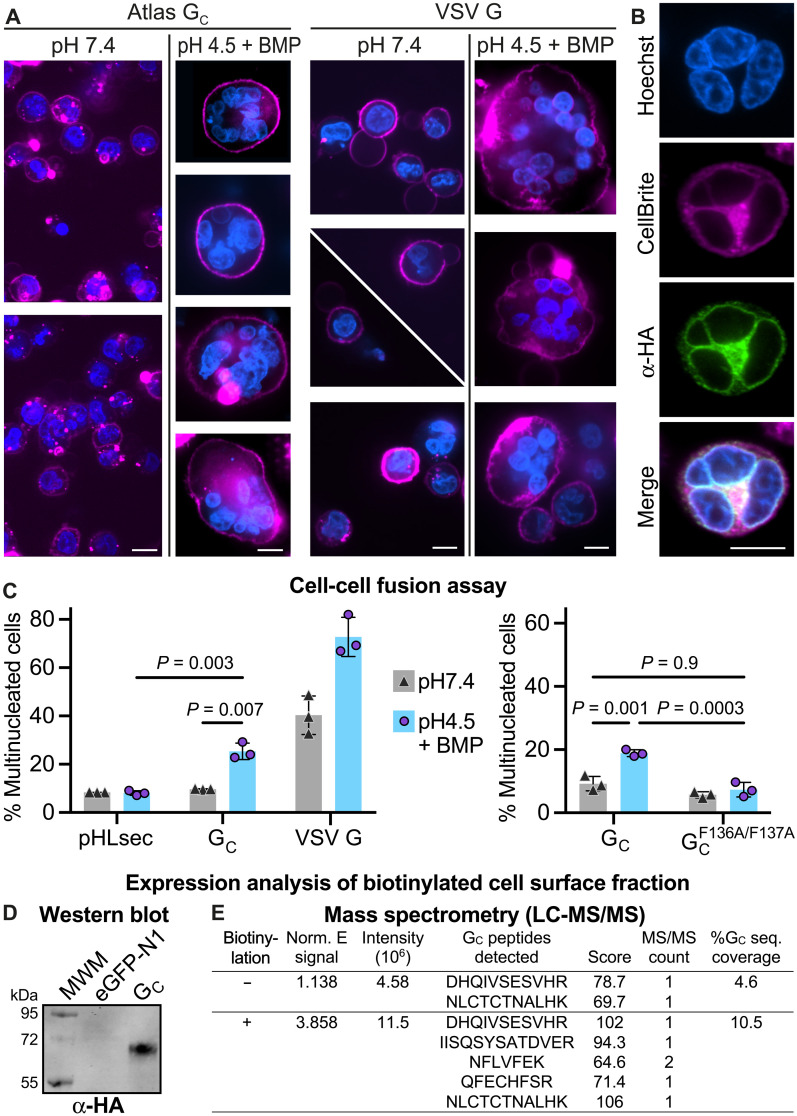
Cell-cell fusion assay. (**A**) Confocal micrographs of CHO cells expressing Atlas G_C_ fused to the transmembrane anchor from HLA-A2 or VSV G. Cells were treated with pH 4.5 buffer containing BMP or pH 7.4 buffer without lipid. Blue, Hoechst 33342 nuclear stain; magenta, CellBrite Red plasma membrane dye. Micrographs were selected to show fusion events—see fig. S9 for representative raw micrographs. Scale bars, 10 μm. (**B**) Confocal micrograph of a multinucleated CHO cell expressing Atlas G_C_ following treatment with pH 4.5 and BMP. Blue, Hoechst 33342; magenta, CellBrite Red; green, α-HA antibody for Atlas G_C_ detection. Scale bar, 10 μm. A composite *Z* stack of this cell is shown in movie S1. (**C**) The fraction of multinuclear cells transfected with plasmids encoding Atlas G_C_, VSV G, or pHLsec empty vector was calculated by counting mono- and multinucleated cells using the Hoechst and CellBrite stains. Error bars represent SD between measurements from three distinct experiments, with 33 to 568 nuclear clusters counted per replicate. *P* values were calculated by two-way analysis of variance (ANOVA) (Tukey’s multiple comparisons test, GraphPad Prism 9). (**D** and **E**) G_C_ cell surface expression analysis. Cells were biotinylated, cell surface proteins were affinity-purified with NeutrAvidin agarose, and the cell surface G_C_ fraction was quantified by Western blot (**D**) and LC-MS/MS (**E**). See dataset S2 for source data.

Having identified the hallmarks of a fusion protein in Atlas G_C_, we measured its membrane fusion activity in a cell-cell fusion assay. Chinese hamster ovary (CHO) cells were transfected with plasmids encoding Atlas G_C_. To promote plasma membrane localization and minimize endoplasmic reticulum retention, we replaced the predicted transmembrane domain and cytosolic tail of G_C_ with the C-terminal transmembrane anchor and cytosolic tail from human leukocyte antigen A2 (HLA-A2), known to localize to the plasma membrane (*67*). Plasmids encoding vesicular stomatitis virus G (VSV G), or no protein, were used as positive and negative controls, respectively. Because Atlas G_C_ trimers require low pH and BMP or PS to efficiently bind membranes, we treated transfected cells with exogenous BMP and then transferred them to pH 4.5 buffered medium to trigger fusion. Similar treatments have been used previously to measure cell-cell fusion activity of flavi- and alphaviruses (*48*). Confocal light microscopy with nuclear and plasma membrane stains showed that cells with three or more nuclei were common in cells expressing Atlas G_C_ following treatment with BMP and pH 4.5, although less abundant than in cells expressing VSV G, with or without treatment (Fig. 6, A and B, and movie S1). To quantify cell-cell fusion, we counted nuclei and multinuclear cells in micrographs. We found that in cells expressing Atlas G_C_, the fraction of multinuclear cells, defined as cells containing two or more nuclei, was 25 ± 3% following treatment with BMP and pH 4.5 versus 10 ± 0.2% without treatment (95% confidence intervals; Fig. 6C). Transfection of the G_C_ fusion loop mutant F136A/F137A resulted in essentially identical multinucleation fractions as with empty vector, with or without treatment with BMP and pH 4.5 (Fig. 6C), underpinning the importance of the fusion loop in fusion activity. By comparison, in cells expressing VSV G, 73 ± 8% of treated cells were multinuclear. In addition, 8 ± 1% of cells transfected with empty vector were binuclear with or without treatment, but none contained more than two nuclei (fig. S9). Incubation of transfected cells with cyclin-dependent kinase 4 (CDK4), which arrests the cell cycle at G_1_ phase, had no measurable effect on G_C_-induced cell multinucleation (fig. S9D), indicating that cytokinesis effects do not significantly contribute to this cell-cell fusion assay readout. We conclude that Atlas G_C_ has approximately one-third of the membrane fusion activity of VSV G under the treatment conditions tested, which is substantial given that VSV G is considered highly fusogenic and widely used as a model fusogen.

To confirm that G_C_ was present on the surface of cells transfected with a plasmid encoding G_C_, cell surface proteins were biotinylated, affinity-purified, and analyzed by immunoblot and liquid chromatography with tandem mass spectrometry (LC-MS/MS). G_C_ was readily detected in these fractions by Western blot (Fig. 6D) and LC-MS/MS (Fig. 6E).

## DISCUSSION

Here, we identify Atlas virus as an endogenous belpaovirus containing intact *gag*, *pol*, and *env* genes with previously unknown features in the human hookworm *A. ceylanicum*. The cryo-EM structure of the Atlas Env reveals a class II viral fusion protein fold similar to that of the G_C_ glycoprotein from RVFV. Atlas G_C_ has the hallmarks of an active class II membrane fusion protein: a stable trimeric assembly, a putative fusion loop, membrane insertion triggered by low pH with specificity for late endosomal lipid composition, and membrane fusion activity.

Our work supports the model first proposed on the basis of phylogenetic studies that the nematode belpaoviruses acquired their *env* by horizontal gene transfer from a virus from the family Phenuiviridae or a phlebovirus-like ancestor (*14*). It remains unclear whether RNA encoding the phlebovirus-like glycoprotein integrated into the belpaovirus ancestor as mRNA in a splicing event or by first becoming a substrate for the RT with subsequent genomic integration as double-stranded DNA. The envelope proteins from retroviruses, including ERVs, that have been biochemically characterized were all found to be class I fusion proteins with an α-helical coiled coil as the core fold. Viral class II fusion proteins have so far been found only in nonintegrating RNA viruses. Our discovery of an endogenous belpaovirus with a potentially functional, phlebovirus-like class II fusion protein that is structurally unrelated to retrovirus Envs reveals an unexpected degree of structural and genetic plasticity in reverse-transcribing RNA viruses. More generally, the presence of the class II fusion protein fold in EVEs and across many phyla suggests that this fold is derived from a common ancestor that could have been viral or cellular in origin (*32*–*35*, *68*).

While rare, horizontal gene transfer of atypical fusogens into retroelements is not unique to the belpaoviruses. The Tas element from the nematode *Ascaris lumbricoides*, a common parasitic worm in humans, has an *env* gene with weak genetic similarity to herpesvirus gB proteins (*14*), which have a class III fusion protein fold (also found in Rhabdoviridae and Baculoviridae). Together, these findings lead us to hypothesize that acquisition of a fusion protein from an infectious virus represents a general paradigm of how retrotransposons can become retroviruses and how ancestral reverse-transcribing viruses may have originated.

The *env* gene is often the first element to be lost in EVEs, as it is not required for intracellular proliferation, so it is notable that the G_N_-G_C_ *env* module is intact in the Atlas virus. With its identical LTRs and no stop codons or frameshift mutations, the Atlas virus shows all the signs of being intact and recently active. This supports the notion that the envelope may be functional (*12*). The preserved biological activities of Atlas G_C_ suggest these activities could have cellular functions in health and disease, as reported for a small but increasing number of ERV *env* and *gag* gene products (*1*, *3*, *5*, *6*). RNA sequencing (RNA-seq) data for *A. ceylanicum* (*69*) suggest that Atlas and some other complete belpaoviruses are transcribed, with transcript abundance varying across developmental stages (fig. S10). A subset of belpaoviruses has also been reported to be highly transcribed in the parasitic trematode *Schistosoma mansoni* (blood fluke) (*70*). Further studies are required to determine the full extent to which protein expression from transposable elements—and its dysregulation—contribute to basic cellular functions, embryonic development, and disease outcomes. This work provides a blueprint for such efforts.

## MATERIALS AND METHODS

### Genomic analyses of *A. ceylanicum* Atlas virus

A PSI-BLAST search for protein sequences similar to biochemically characterized phlebovirus fusion proteins identified the gene *Acey_s0020.g108* (UniProt: A0A016UZK2; GenBank: JARK01001356.1; genomic translation EYC20859.1) in the human hookworm *A. ceylanicum* as the most similar sequence outside infectious virus taxa, with an *E* value of 10^−20^ against the RVFV G_C_ sequence (UniProt: P03518). A second iteration performed using position-specific scoring matrix based on an alignment of sequences identified in the first iteration gave an *E* value of 10^−144^. The *Acey_s0020.g108* gene is referred to here as the Atlas virus.

Phylogenetic analysis of the phlebovirus G_C_-like protein from the Atlas virus and other G_C_ or G_C_-like proteins with similar protein sequences was performed as follows. A reference multiple-sequence alignment was initially generated by aligning the Atlas G_C_ protein sequence with the sequences of reference protein RVFV G_C_ and other viral G_C_ proteins identified as having structural similarity in structure comparisons with Dali (*71*), namely, the G_C_ proteins from DABV (formerly SFTSV; GenBank: AGM33042.1) and HRTV (GenBank: AFP33394.1). For each G_C_ protein, the sequence range present in the corresponding Protein Data Bank (PDB) entry (7A4A, 6EGU, 5G47, or 5YOW, respectively) was defined as the G_C_ ectodomain sequence and used to generate the reference alignment with MAFFT in SnapGene v5.1.7. The sequences of the following EVEs with detectable Env protein sequence similarity were then added to the alignment: *Necator americanus* NECAM_13468 (GenBank: XM_013440329.1), *C. elegans* Cer13 virus (GenBank: Z81510.2; WormBase, WBTransposon00000728, gene Y75D11A.5), and seven *A. ceylanicum* EVEs encoding complete Gag-Pol-Env polyproteins with predicted phlebovirus-like Envs (Fig. 1B; GenBank: EYC27361.1, EYC19962.1, EYC20099.1, EYC18998.1, EYC07469.1, EYB91703.1, and EYB80846.1). The tree was calculated with IQ-TREE v1.6.12 and drawn with iTOL v6.

The RT phylogenetic tree was calculated on the basis of the protein sequences of Atlas virus, Cer13 virus, the seven *A. ceylanicum* EVEs listed above, and seven other belpaoviruses (GenBank: AF060859.1, Z29712.1, L09635.1, U23420.1, AY180917.1, D83207.1, and AF537216.1). RT domains (RVT_1; Pfam: PF00078) were identified with PfamScan in five of the sequences (GenBank: Z81510.2, EYC27361.1, EYC19962.1, EYC07469.1, and EYB80846.1). The protein sequences of these five RTs were extracted and aligned with MAFFT and Clustal Omega v1.2.4. The tree was generated with IQ-TREE v1.6.12 and iTOL v6. The Atlas virus LTRs were identified, and sequence identity matrices calculated as described in fig. S1.

### Protein expression and purification

Synthetic genes encoding soluble ectodomain fragments of the Env of ERV Y032_0020g108 from *A. ceylanicum* were subcloned into the pMT/BiP/V5-His vector (Thermo Fisher Scientific) under the metallothionein (MT) promoter and in frame with the immunoglobulin heavy-chain binding protein (BiP) signal sequence and the C-terminal V5 and six histidine tags. The constructs referred to here as Atlas G_C_ and Atlas G_C_(DI-III) span amino acids 2330 to 2772 and 2330 to 2751 from UniProt (A0A016UZK2), respectively. Atlas G_C_ mutants were generated by Dpn I–based site-directed mutagenesis. D.mel-2 insect cells (Thermo Fisher Scientific) were cotransfected with the expression construct and blasticidin resistance marker pCoBlast (Thermo Fisher Scientific) at a 20:1 molar ration and cultured for 6 weeks in blasticidin (0.5 μg ml^−1^) to obtain a population of expressor cells. Expression was induced in a shaking cell suspension at 27°C with 0.5 mM CuSO_4_ at a cell density of 5 × 10^6^ cells ml^−1^. The cell culture medium was harvested 4 to 5 days after induction, centrifuged to remove cells (2000*g*) and cell debris (17,000*g*), filtered with a 0.2-μm filter, concentrated by tangential flow filtration, and buffer-exchanged into 20 mM tris (pH 7.8), 0.3 M NaCl, 5% glycerol, 20 mM imidazole, and 0.5 mM TCEP [tris(2-carboxyethyl)phosphine]. Atlas G_C_ was purified by nickel affinity chromatography with a HisTrap Excel column (Cytiva), followed by anion exchange chromatography with a MonoQ or Resource Q column (Cytiva) using 20 mM tris (pH 8.0), 50 mM NaCl, 5% glycerol, and 0.5 mM TCEP as the binding buffer and binding buffer plus 1 M NaCl as the elution buffer. Peak fractions were concentrated and further purified by size exclusion chromatography (SEC) with a Superdex 200 Increase (10/300) column (Cytiva) in 20 mM tris (pH 7.8 to 8.0), 0.15 M NaCl, 5% glycerol, and 0.5 mM TCEP. The C-terminal V5 and histidine tags were optionally cleaved by incubation with carboxypeptidase A (CPA) for 3 hours at 4°C (1:500 CPA:G_C_ molar ratio).

### Liposome binding assay

1-Palmitoyl-2-oleoyl-*sn*-glycero-3-phosphatidylcholine (PC), 1-palmitoyl-2-oleoyl-*sn*-glycero-3-phosphatidylethanolamine (PE), egg SM, 1-palmitoyl-2-oleoyl-*sn*-glycero-3-phosphatidyl-l-serine (PS), 1-palmitoyl-2-oleoyl-*sn*-glycero-3-phospho-(1′-rac-glycerol) (PG), (*S*,*R*) bis(monoacylglycero)phosphate (BMP) (Avanti Polar Lipids), and 1-cholesterol (Sigma-Aldrich) were dissolved in chloroform. Lipid solutions (25 mM) were mixed at various molar ratios and dried under nitrogen gas for more than 4 hours. The lipid film was resuspended in liposome buffer [20 mM tris (pH 7.8), 0.15 M NaCl, 5% glycerol, 0.5 mM TCEP, 2 mM MgCl_2_, 2 mM CaCl_2_, and 2 mM KCl] and subjected to five cycles of freeze-thawing in liquid nitrogen, followed by 25 cycles of extrusion through two 0.2-μm polycarbonate filter membranes (Whatman). Purified Atlas G_C_ ectodomain was added in a 1:771 protein:lipid molar ratio and incubated at 37°C for 5 min. The pH was reduced by adding a 2 M stock solution of sodium acetate (pH 4.6) or 4.0 to a final concentration of 0.2 M. Following a 2-hour incubation at 37°C, the pH of the suspension was neutralized with 1 M tris (pH 8). OptiPrep density gradient medium (Sigma-Aldrich) was added to a concentration of 40%, maintaining 0.15 M NaCl throughout. Approximately 0.5 ml of the liposome suspension was placed in a centrifuge tube, overlaid with a 2.5-ml cushion of 30% OptiPrep solution, and centrifuged at 100,000*g* for 1 hour at 4°C in a TLA100.3 rotor (Beckman Coulter). Top and bottom fractions (approximately 1.5 ml each) were collected from the top meniscus with a micropipette. Atlas G_C_ was quantified by densitometry of the absorbance at 700 nm of bands in Coomassie-stained SDS–polyacrylamide gel electrophoresis (PAGE) gels with an Odyssey scanner (LI-COR). Flotation was defined as the amount of Atlas G_C_ ectodomain in the top fraction divided by the total amount of Atlas G_C_ ectodomain in both fractions.

For measurement of liposome diameter by DLS, the liposome suspensions were diluted 10-fold in liposome buffer before the addition of 40% OptiPrep solution. Following centrifugation, liposome diameters were measured in 384-well clear-bottomed optical imaging plates (Corning) with a DynaPro Plate Reader III (Wyatt Technologies). The mean diameter was calculated as the average of three independent measurements, each consisting of 15 2-s acquisitions. Protein-free acidified liposome controls were treated and measured in parallel, with the liposome buffer instead of the Atlas G_C_ solution.

### SEC and multiangle scattering analysis

Samples (100 μl) containing Atlas G_C_ ectodomain (1.6 to 2.5 mg ml^−1^) were analyzed by SEC at 293 K on a Superdex 200 (10/300) column (Cytiva) in 20 mM tris (pH 7.8), 0.15 M NaCl, 5% glycerol, and 0.5 mM TCEP with a flow rate of 0.5 ml min^−1^. The SEC system was coupled to both multiangle light scattering and quasi-elastic light scattering modules (DAWN-8+, Wyatt Technology). The protein was also detected as it eluted from the column with a differential refractometer (Optilab T-rEX, Wyatt Technology) and an ultraviolet (UV) detector at 280 nm (Agilent 1260 UV, Agilent Technology). Molar masses of peaks in the elution profile were calculated from the light scattering and protein concentration, quantified using the differential refractive index of the peak assuming a specific refractive index increment, dn/dc, of 0.1860, with ASTRA6 (Wyatt Technology).

### Cryo-EM sample preparation and data collection

Purified Atlas G_C_ ectodomain trimer (3 μl at a concentration of 0.025 mg ml^−1^) in 20 mM tris (pH 7.8), 0.15 M NaCl, 5% glycerol, and 0.5 mM TCEP was applied onto glow-discharged R1.2/1.3 400 mesh copper grids (Quantifoil Micro Tools, Germany). The grids were blotted for 4 s and plunge-frozen in liquid ethane with a Vitrobot Mark IV (Thermo Fisher Scientific) at 4°C and 100% humidity. Preliminary sample screening and initial datasets were acquired on a FEI Tecnai F20 microscope operated at 200 kV equipped with a Falcon II direct electron detector (Thermo Fisher Scientific) at −4-μm defocus. High-resolution cryo-EM dataset collection was performed on a Titan Krios microscope (Thermo Fisher Scientific) operated at 300 kV equipped with a 20 eV slit-width GIF Quantum energy–filtered Gatan K2 Summit direct electron detector in counting mode. A total of 3027 movies were recorded at a calibrated magnification of ×130,000, leading to a magnified pixel size of 1.047 Å on the specimen. Each movie comprised 36 frames with an exposure rate of 1.28 e^−^ Å^−2^ per frame, with a total exposure time of 8 s and an accumulated exposure of 46.18 e^−^ Å^−2^. Data acquisition was performed with the EPU software for automated data acquisition for single-particle analysis (Thermo Fisher Scientific) with three shots per hole at −1.3- to −3.5-μm defocus.

### Image processing

Micrographs from initial datasets allowed us to obtain a consistent model at ~19 Å resolution from 3790 particles selected after two-dimensional (2D) and 3D classification, and consequent autorefinement. All movies from high-resolution datasets were motion-corrected and dose-weighted with MOTIONCOR2 (*72*). Aligned, non–dose-weighted micrographs were then used to estimate the contrast transfer function (CTF) with the program GCTF (*73*). All subsequent image processing steps were performed using RELION 3.0 (*74*). 2D references from initial datasets were used to autopick the micrographs. One round of reference-free 2D classification was performed to produce templates for better reference-dependent autopicking, resulting in a total of 987,570 particles. After a first round of 2D classification, 595,011 particles were selected to perform a second 2D classification, resulting in a final number of 320,041 selected particles. Then, a 3D classification imposing C3 symmetry was performed using the model from the initial datasets filtered at 40 Å resolution as the initial model. The best class, containing 197,145 particles, was selected and subjected to 3D autorefinement imposing C3 symmetry, yielding a map with an overall resolution at 4.11 Å based on the gold standard [Fourier shell correlation (FSC) = 0.143] criterion. After refinement, the CTF refinement (per-particle defocus fitting and beam tilt estimation) and Bayesian polishing routines implemented in RELION 3.0 were performed, yielding a final map with an overall resolution at 3.76 Å. Local resolution was estimated with RELION.

### Model building and refinement

The most similar sequence to Atlas G_C_ with a structure available was glycoprotein G_C_ from RVFV. The crystal structure of RVFV G_C_ in the postfusion conformation [PDB: 6EGU (*27*)] was used as template to build a homology model with the sequence of Atlas G_C_ using the SWISS-MODEL server (https://swissmodel.expasy.org). The output model was docked as a rigid body into the density with UCSF Chimera (*75*). Initial docking was performed manually and was followed by real-space fitting with the Fit in Map routine. A preliminary step of real-space refinement was performed on the three-subunit model, with Phenix 1.13 (*76*), with global minimization, atomic displacement parameter (ADP), simulated annealing, and morphing options selected. The model was then rebuilt in Coot (*77*) to optimize the fit to the density. Because of low-resolution information in the fusion loop region, the density was converted to .mtz file using CCP-EM software package tools, and blurring of the density allowed us to localize bulky residues and disulfide bonds and thus use them as a guide to build the entire fusion loop. A final step of real-space refinement was performed with Phenix 1.15, with global minimization and ADP options selected. The following restraints were used in the real space refinement steps: secondary structure restraints, noncrystallographic symmetry restraints between the protein subunits, side chain rotamer restraints, and Ramachandran restraints. Key refinement statistics are listed in table S1.

### Model validation and analysis

The FSC curve between the final model and full map after postprocessing in RELION, model versus map, is shown in fig. S3A. Cross-validation FSC curves (fig. S3B) were calculated as follows. The atoms in the final atomic model were displaced by 0.5 Å in random directions with Phenix. The shifted coordinates were then refined against one of the half-maps generated in RELION, the “work set.” This test refinement was performed in Phenix using the same procedure as for the refinement of the final model (see above). The other half-map, the “test set,” was not used in refinement for cross-validation. FSC curves of the refined shifted model against the work set, FSCwork, and against the test set, FSCtest, are shown in fig. S3. The FSCwork and FSCtest curves are not substantially different, consistent with the absence of overfitting in our final models. The quality of the atomic models, including basic protein geometry, Ramachandran plots, and clash analysis, was assessed and validated with Coot, Phenix 1.15, and the Worldwide PDB (wwPDB) OneDep System (https://deposit-pdbe.wwpdb.org/deposition).

### Cell-cell fusion assay

CHO Lec3.2.8.1 cells were transfected with pHLsec plasmids encoding ectodomain fragments of Atlas G_C_ (residues 2330 to 2795) fused to the C-terminal transmembrane domain from HLA-A2 (residues 288 to 345) and cloned in frame with the vector’s secretion signal and a C-terminal hemagglutinin (HA) tag. Empty pHLsec plasmid and pcDNA encoding VSV G were used as negative and positive controls, respectively. Sixteen to 20 hours after transfection, cells were transferred to phosphate-buffered saline (PBS) supplemented with 2.5 mM BMP [18:1 (*S*,*S*) bis(monoacylglycero)phosphate; Avanti Polar Lipids]. To obtain a homogeneous BMP suspension, the mixture was freeze-thawed five times using liquid nitrogen and a water bath, followed by a 3-min incubation in a sonicating water bath. Cells were incubated in the BMP suspension (or PBS for the untreated control) at 37°C for 5 min, shown previously to be sufficient for anionic lipid incorporation into the plasma membrane (*48*). Cells were transferred to pH 4.5 complete medium [Dulbecco’s modified Eagle medium adjusted to pH 4.5 with HCl supplemented with 10% fetal bovine serum (FBS)] or pH 7.4 complete medium for the untreated control and centrifuged at 2500*g* at 37°C for 2 min. Cells were immediately resuspended in complete media and plated out. Following reattachment, 4 to 6 hours after treatment, cells were washed with PBS, fixed with 4% formaldehyde for 5 to 10 min, and washed three times with PBS. Cell were then stained with Hoechst 33342 (Bio-Rad) and CellBrite Red cytoplasmic membrane dye (Biotium, catalog no. 30023) and imaged on a Nikon iSIM Swept Field inverted confocal microscope with a 60×/1.2–numerical aperture (NA) water objective.

To control for the contribution of the cell cycle to Atlas-G_C_–induced multinucleation, we repeated the cell-cell fusion assay in the presence of the cell cycle inhibitor CDK4. The assay was performed as described above, except for the following modifications: The assay was performed with human embryonic kidney (HEK) 293 T cells; BMP lipid was added 36 to 40 hours after transfection; 10 μM CDK4 (Cayman Chemical, catalog no. 17648) was included in the medium used to resuspend the cells after centrifugation at pH 4.5; after staining with Hoechst 33342 and CellBrite Red, cells were stained overnight at 4°C with anti-CD98 mouse monoclonal immunoglobulin G_1_ κ (Santa Cruz Biotechnology, sc-376815; 1:100 dilution in 20% FBS), followed by Alexa Fluor 568 secondary antibody (Thermo Fisher Scientific, A11004; RRID:AB_ 2534072; 1:500 dilution in 20% FBS for 1 hour); and cells were imaged on a Zeiss 780 inverted confocal microscope with a 40×/1.3-NA oil objective or a Nikon CSU-W1 Spinning Disk inverted confocal microscope with a 60×/1.2-NA water objective.

For most images, cluster analysis of the Hoechst channel was used to count single nuclei and identify polynuclear clusters (fig. S9). For a subset of images with small numbers of nuclei, mono- and polynuclear clusters were counted manually. Nuclei within polynuclear clusters were counted by visual inspection. The plasma membrane stain was used to confirm polynuclear clusters and count the number of multinuclear cells, defined as cells with two or more nuclei, by visual inspection. For some images containing large syncytia, due to poor plasma membrane staining of the syncytia, the Hoechst channel was used to count the total number of nuclei, and the CellBrite and Alexa Fluor 568 channels were used to manually count mononucleated cells, which had clearly distinguishable plasma membranes. The fraction of multinucleated cells (*F*) was calculated using the formula

F=1−(n.mononucleated cells)/(total n.nuclei)

### Cell surface biotinylation and MS

Proteins on the cell surface were biotinylated and isolated with the Pierce Cell Surface Protein Biotinylation and Isolation Kit (Thermo Fisher Scientific, A44390). HEK293T cells were transfected with 3 μg of pHLsec-G_C_ or enhanced green fluorescent protein–N1 (control) plasmid. One day later, cells were washed with PBS and incubated with sulfo-NHS-SS-biotin (0.25 g/liter) on ice for 10 min. Then, cells were washed with PBS and suspended in 0.5 ml of Thermo Fisher Scientific lysis buffer supplemented with cOmplete EDTA-free Protease Inhibitor Cocktail (Merck). Cells were lysed on ice for 30 min (with 5-s vortexing before and after lysis) and centrifuged at 15,000*g* for 5 min at 4°C. The lysate supernatant was incubated with 0.25 ml of NeutrAvidin Agarose resin for 30 min at 20°C on an end-over-end rotator. The resin was washed with wash buffer, and bound proteins were eluted with elution buffer from the kit.

Samples were prepared for MS with the EasyPep Mini MS Sample Prep Kit (Thermo Fisher Scientific, A40006) following the manufacturer’s instructions. Briefly, proteins were extracted, reduced, alkylated, and digested with trypsin/Lys-C protease. Hydrophilic and hydrophobic contaminants were removed with a peptide cleanup step. LC-MS/MS analysis of peptide samples was performed on an Ultimate 3000 rapid separation liquid chromatography system connected to a Q-Exactive plus mass spectrometer (Thermo Fisher Scientific). The acquired raw files were processed with MaxQuant v1.6.6.0.

### Western blotting

The cell surface biotinylated protein samples purified from HEK293T cells as described above were heated to 95°C in SDS sample buffer for 10 min. A total of 15 μl of each sample was run on a polyacrylamide gel. Gels were blotted onto polyvinylidene difluoride membranes (Merck). Blots were blocked in 5% milk in PBS and 0.2% Tween 20 and incubated overnight with anti-HA rabbit monoclonal antibody (Cell Signaling Technology, 3724; RRID:AB_1549585) diluted 1:1000 in blocking solution. Blots were imaged with the 800-nm channel of a LI-COR Odyssey fluorescent scanner after incubation with anti-rabbit DyLight 800–conjugated secondary antibody (Cell Signaling Technology, 5151; RRID:AB_10697505) at 1:5000 dilution for 30 min at room temperature.

### RNA-seq analysis

We analyzed data published by Hawdon and colleagues (*69*) (fig. S10). ArrayExpress run accession numbers and corresponding life cycle stages were as follows: SRR6359160, L3; SRR6359161 and SRR6359163, L4; SRR6359164 and SRR6359165, adult mixed pooled worms; and SRR6359162 and SRR6359166, adult male pooled worms. Our mapping and counting strategy was modified as recommended for repetitive genomic features (*78*). Reads were mapped against the reference *A. ceylanicum* genome (*79*) with STAR v2.7.5a with parameters: “--outSAMtype BAM SortedByCoordinate --runMode alignReads --outFilterMultimapNmax 1000 --outSAMmultNmax 1 --outFilterMismatchNmax 3 --outMultimapperOrder Random --winAnchorMultimapNmax 1000 --alignEndsType EndToEnd --alignIntronMax 1 --alignMatesGapMax 350.” FeatureCounts v2.0.1 with parameters “-M -F GFF -s 0 -p -t exon -g gene_id” was used to count reads over a modified annotation file based on the original annotation (*79*) and downloaded from Wormbase Parasite (https://parasite.wormbase.org). The original annotation file in GFF format was slightly modified to contain a “gene_id” feature in the ninth column and hence facilitate the calculation of aggregate reads by gene (see header of the GFF file, dataset S3). RNA-seq analysis scripts and instructions of how to use them are provided in the Supplementary Materials (dataset S4) and are additionally available on Github (https://github.com/annaprotasio/Merchant_et_al_2020).

### Statistics

Error bars represent the SD or SE—as indicated in the respective figure legend—of two to seven replicates conducted across at least two independent experiments. SDS-PAGE gels and DLS data shown are representative of at least two independent experiments. Significance and *P* values were determined by two-way analysis of variance (ANOVA). For DLS data, Sidak’s multiple comparisons test was used with a 95% confidence interval, in Prism 8 (GraphPad). For cell-cell fusion data, Tukey’s multiple comparisons test was used in Prism 9 (GraphPad). Source data are provided in datasets S1 and S2. No statistical methods were used to predetermine sample size, experiments were not randomized, and the investigators were not blinded to experimental outcomes.

## Supplementary Materials

### This PDF file includes:

Figs. S1 to S10 Table S1 References
