# Supplementary material for: A bioactive phlebovirus-like envelope protein in a hookworm endogenous virus
Source: Sci Adv. 2022 May 11;8(19):eabj6894. doi: 10.1126/sciadv.abj6894 (PMC9094657; doi:10.1126/sciadv.abj6894)
Supplement: Supplementary file 1 — Figs. S1 to S10 Table S1 References [file sciadv.abj6894_sm.pdf]

Science Advances
AAAS

Supplementary Materials for

A bioactive phlebovirus-like envelope protein in a hookworm endogenous virus

Monique Merchant, Carlos P. Mata, Yangci Liu, Haoming Zhai, Anna V. Protasio, Yorgo Modis*

*Corresponding author. Email: ymodis@mrc-lmb.cam.ac.uk

Published 11 May 2022, *Sci. Adv.* **8**, eabj6894 (2022)
DOI: 10.1126/sciadv.abj6894

The PDF file includes:

Figs. S1 to S10
Table S1
Legend for movie S1
Legends for data files S1 to S4
References

Other Supplementary Material for this manuscript includes the following:

Movie S1
Data files S1 to S4

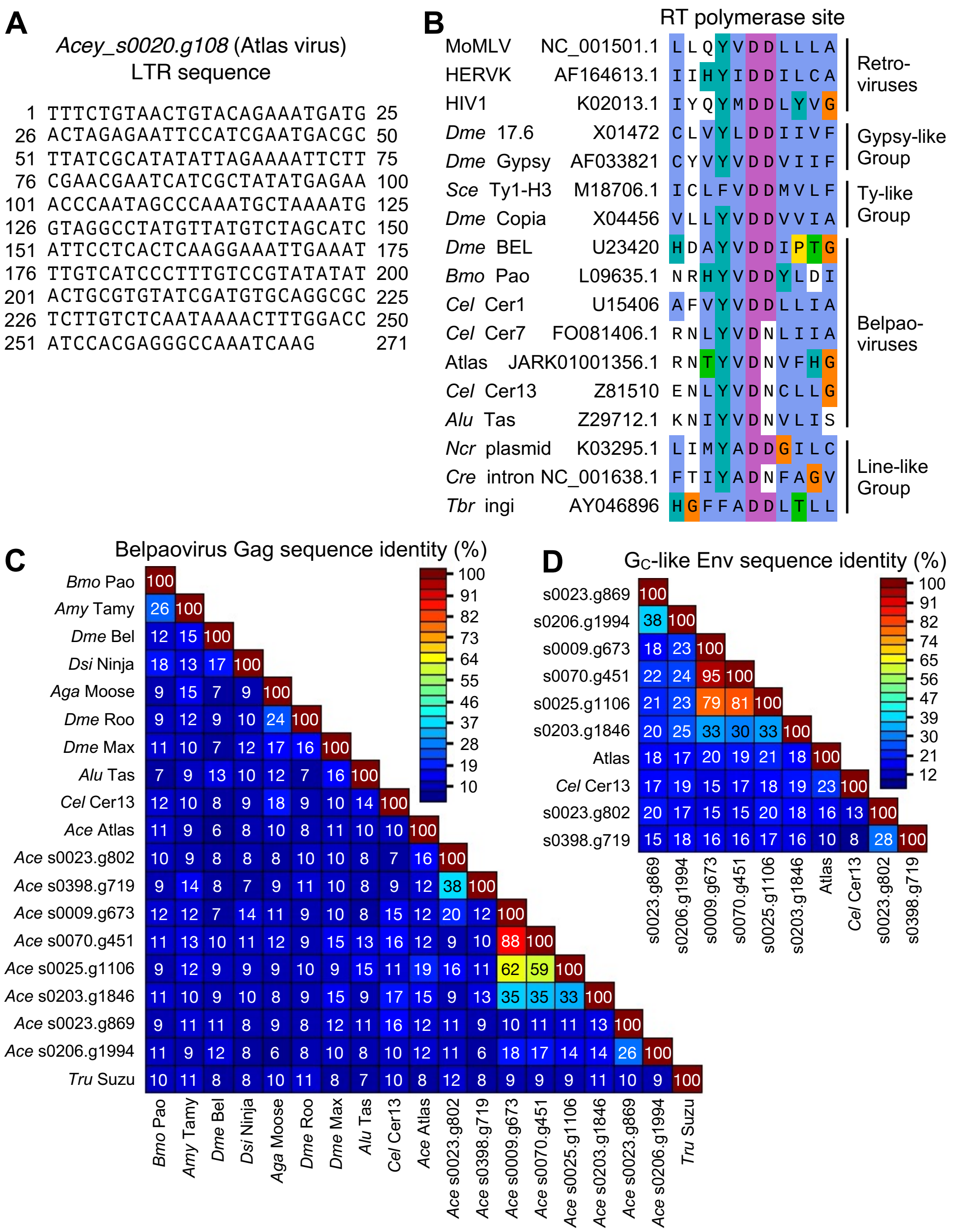

Fig. S1. Atlas is an endogenous belpaovirus with a phlebovirus-like Env.

(**A**) Nucleotide sequence of the Atlas LTRs, predicted by REPuter (*80*). The LTRs are 100% identical. (**B**) Aligned protein sequences of reverse transcriptase (RT) polymerase site from Atlas and other representative belpao- and retroviruses. *A. ceylanicum* belpaoviruses have an Asp to Asn substitution (Y[X]DD to YVDN) in the polymerase site, the most conserved RT motif (Motif V, or Motif C). GenBank accession numbers are listed after virus names. MoMLV, Moloney murine leukemia virus; HERVK, human ERV-K; *Dme*, *Drosophila melanogaster*; *Sce, Saccharomyces cerevisiae*; *Bmo, Bombyx mori*; *Cel, C. elegans*; *Ace, A. ceylanicum*; *Alu, Ascaris lumbricoides*; *Ncr, Neurospora crassa*; *Cre, C. reinhardtii*; *Tbr, Trypanosoma brucei*. (**C**) Sequence identity matrix for Gag protein sequences from representative belpaoviruses and *A. ceylanicum* belpaoviruses with intact protein coding regions. *Amy, Antheraea mylitta; Dsi, Drosophila simulans; Aga, Anopheles gambiae; Tru, Takifugu rubripes.* (**D**) Sequence identity matrix for phlebovirus-like Env protein sequences from nematode belpaoviruses. Identity matrices were calculated with SDT (*81*).

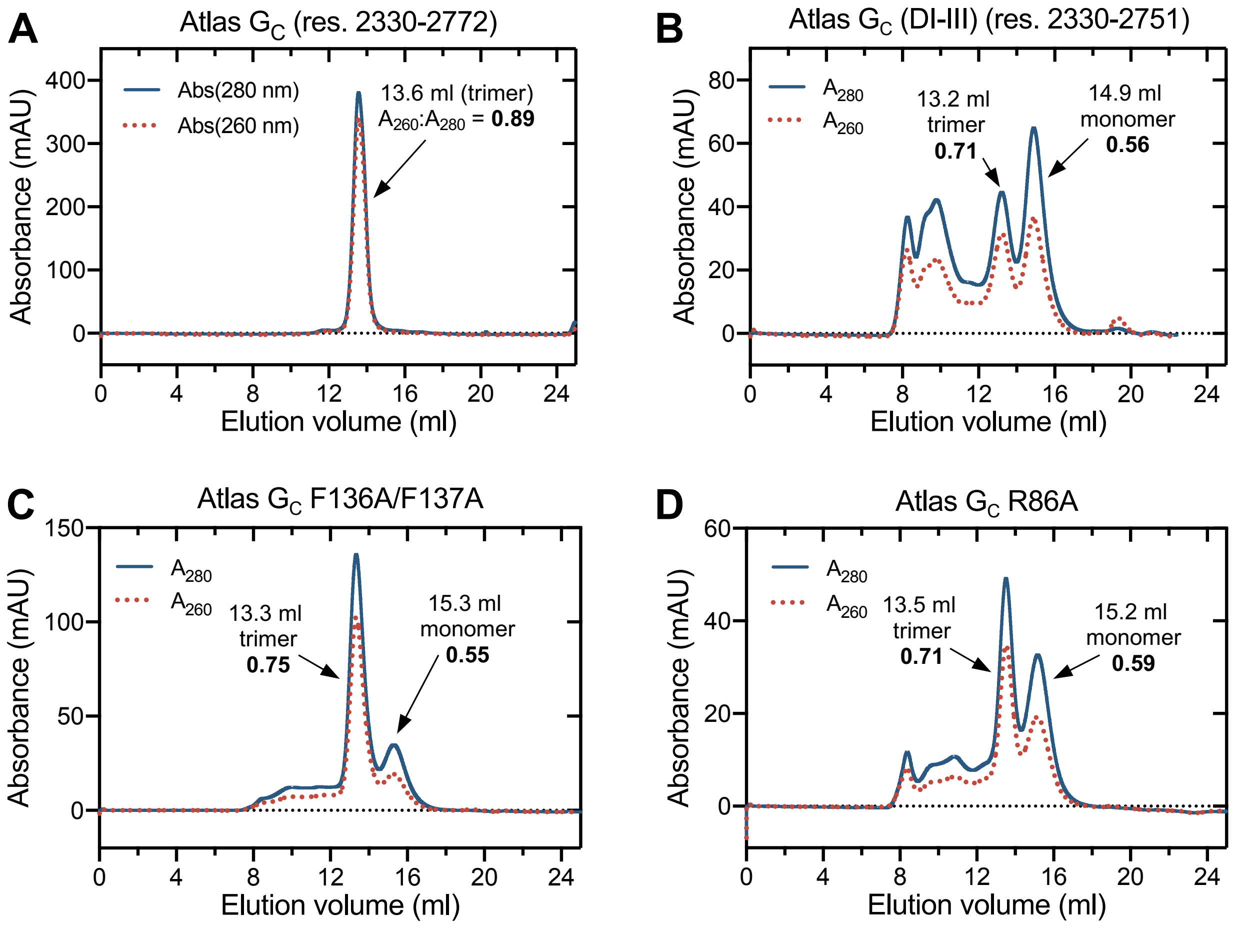

Fig. S2. Size-exclusion chromatography of Atlas G_C following ion-exchange chromatography.

Samples were analysed on a Superdex 200 Increase (10/300) column (Cytiva). The A_{260}:A_{280} ratios for selected peaks are shown in bold. (**A**) The Atlas G_C fragment used for cryo-EM image reconstruction (residues 2330-2772). The protein had higher absorbance at 260 nm (A_{260}) than expected for pure protein. The elution volume was consistent with a homotrimer. (**B**) Atlas G_C(DI-III), maintained at pH > 7 throughout expression and purification. Multiple oligomeric states were present including peaks with elution volumes consistent with monomers, trimers, and higher order oligomers. (**C**) Atlas G_C fusion loop mutant F136A/F137A. (**D**) Atlas G_C GPL binding pocket mutant R86A.

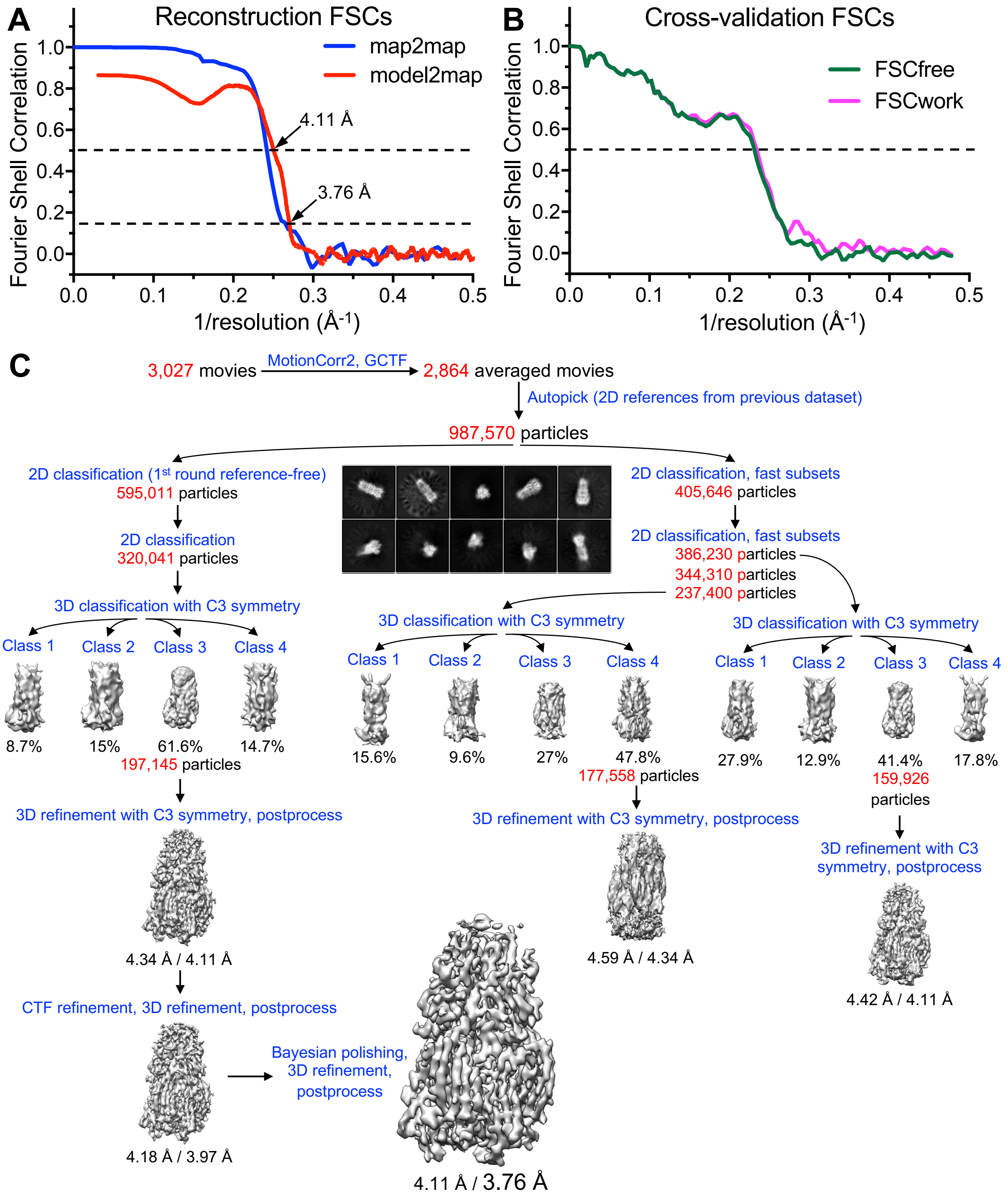

Fig. S3. Image reconstruction quality assessment and workflow.

(**A**) Fourier Shell Correlations (FSC) of the reconstruction of Atlas Gc from two independently refined half-maps (map2map, in blue); and of the reconstruction from the whole dataset versus a map calculated from the refined atomic model (model2map, in red). The gold-standard cutoff (FSC = 0.143) and the FSC = 0.5 level are marked with dashed lines. The resolution values of each curve at these levels are indicated. (**B**) FSC plots for cross-validation as described (*82*). FSCwork (magenta), FSC of refined test model versus work set (half-map used in test refinement). FSCfree (green), FSC of refined test model versus test set (half-map not used in test refinement). The FSC = 0.5 level is indicated by a dashed line. (**C**) Flow chart showing the workflow pipeline for cryo-EM image processing, classification, and model refinement, as described in the Materials and Methods.

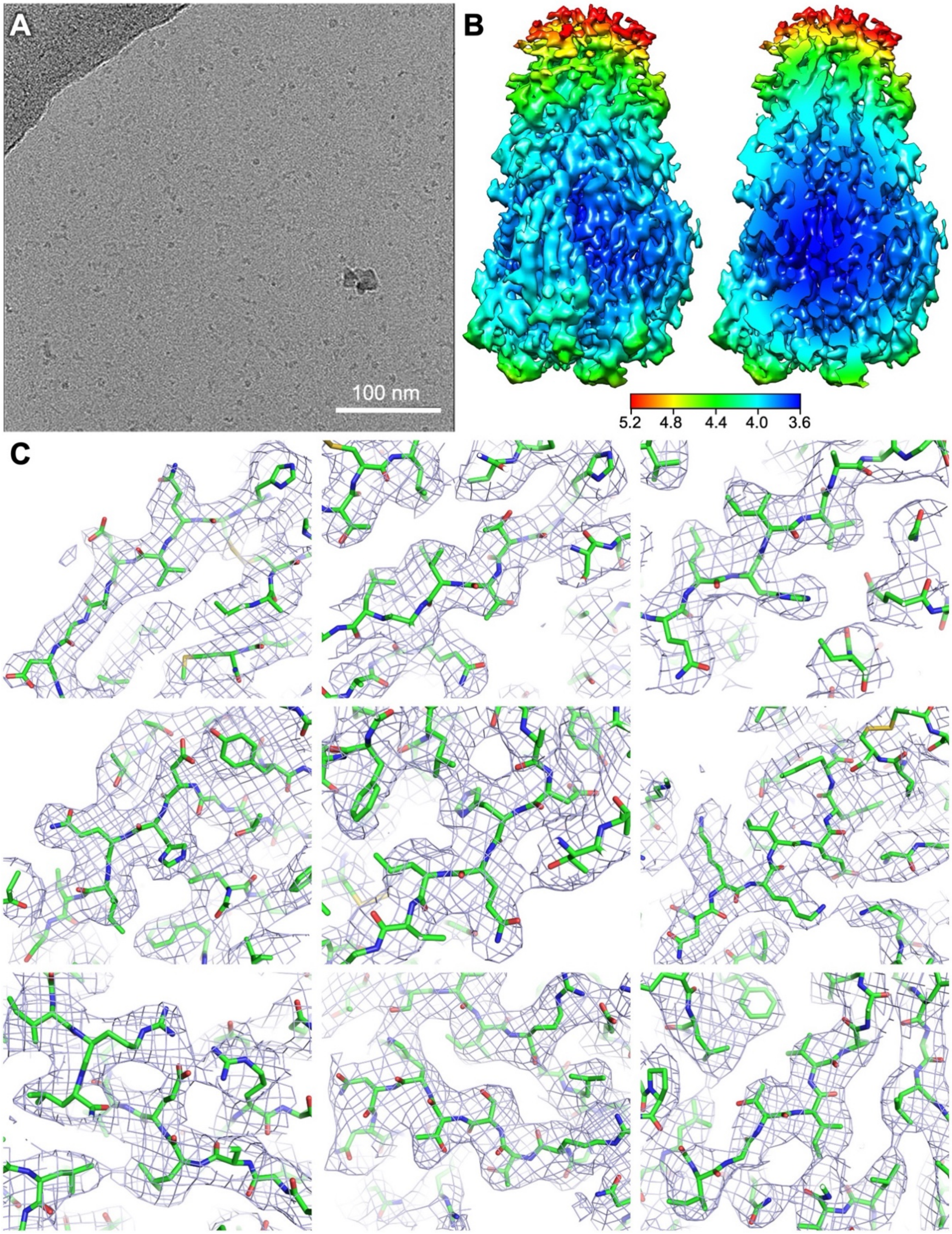

Fig. S4. Representative Cryo-EM micrograph and density with local resolution estimation. (**A**) Representative cryo-EM micrograph of Atlas G_C trimers. (**B**) Local resolution estimation for the cryo-EM volume, calculated in RELION 3.0 (*85*). (**C**) Representative samples of local cryo-EM density from the structure with fitted and refined atomic models. The deposited density map was contoured at 3 σ in Pymol (Schrodinger, LLC).

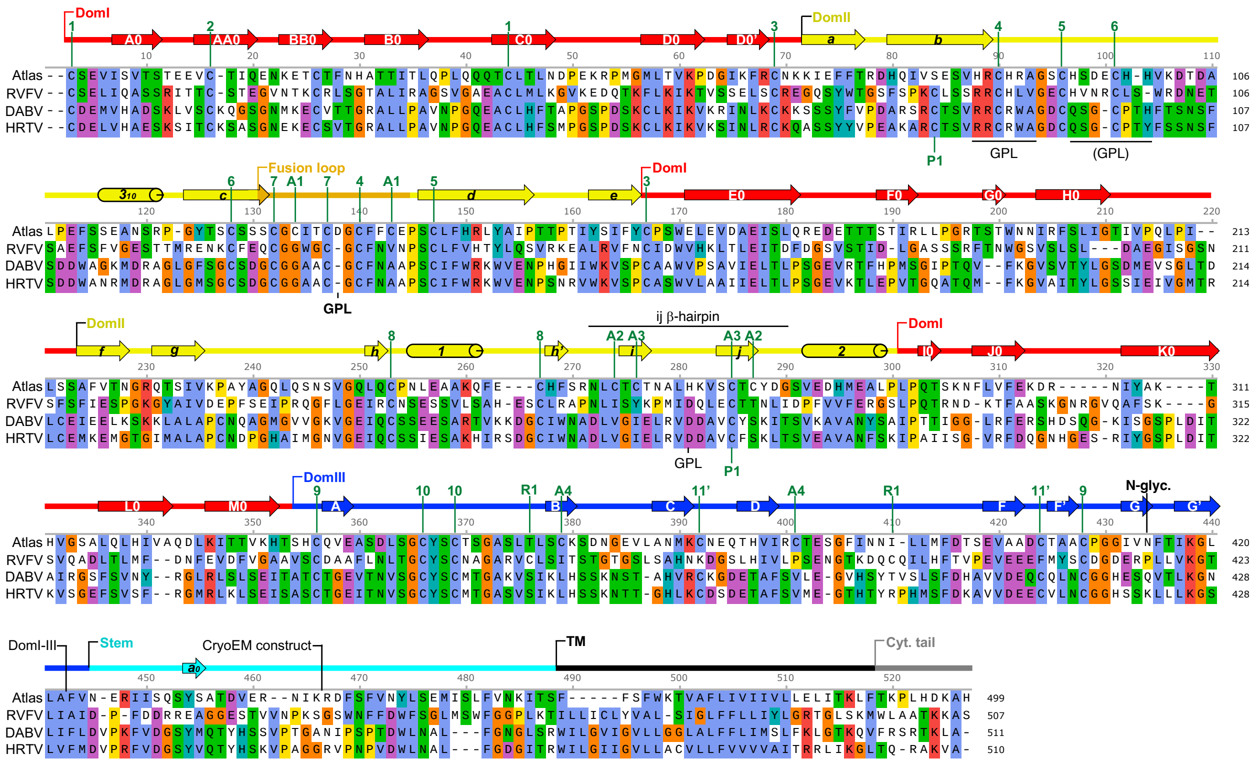

Fig. S5. Protein sequence alignment of Atlas G$_C$ and its closest orthologs.

Amino acid sequence alignment of Atlas G$_C$ and the most similar sequences from infectious viruses: Rift Valley Fever virus (RVFV), Dabie bandavirus (DABV, formerly SFTSV phlebovirus) and Heartland virus (HRTV). Domains, secondary structure elements of Atlas G$_C$, and structural features are marked above the alignment. GPL, residues in or near the glycerophospholipid headgroup binding pocket. N-glyc., N-glycosylation site. Cyt. tail, cytoplasmic tail. Conserved disulfide bonds are numbered in green. Disulfide bonds specific to Atlas virus are denoted with an “A”. An RVFV-specific disulfide is denoted with an “R”. A phlebovirus-specific disulfide is denoted with a “P”. The disulfide marked 11’ is conserved in Atlas, DABV and HRTV but not RVFV.

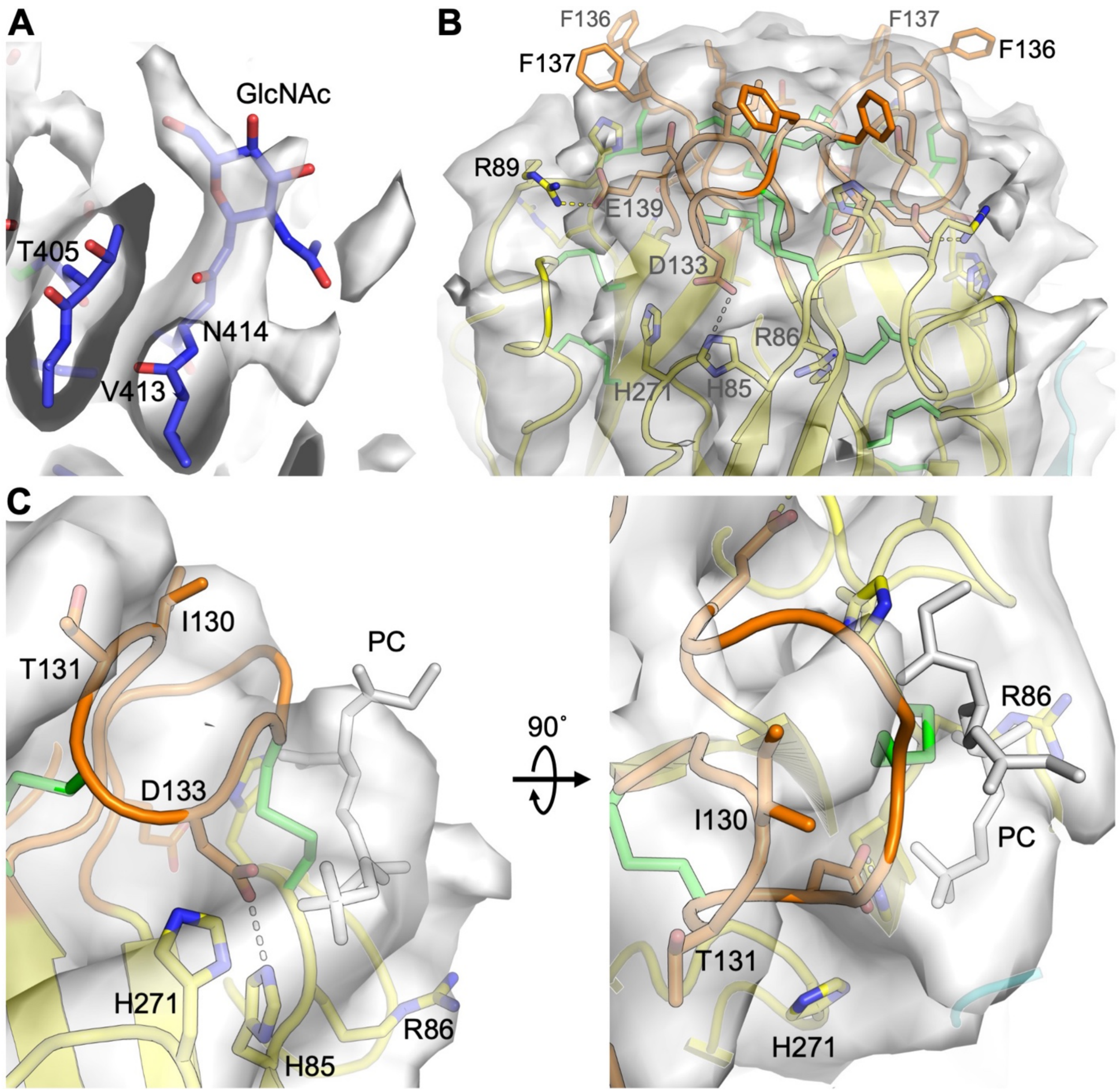

Fig. S6. Cryo-EM density for N-linked glycan and GPL pocket of Atlas G_C.

(**A**) Cryo-EM density and atomic model of the N-linked glycan at Asn414. The deposited density map was contoured at 2.2 σ in Pymol (Schrodinger, LLC). (**B**) Cryo-EM density and atomic model for the fusion loop region of the Atlas G_C trimer. A B-factor blurring correction of +150 $Å^2$ was applied to the deposited density map. The resulting map was contoured at 1 σ in Pymol. (**C**) Cryo-EM density in the glycerophospholipid (GPL) headgroup binding pocket unaccounted for by the atomic model. Shown in grey stick representation is the phosphatidylcholine (PC) headgroup bound to the RVFV G_C structure (*27*) superimposed on the Atlas G_C structure. A B-factor blurring correction of +163 $Å^2$ was applied to the deposited density map. The resulting map was contoured at 1.2 σ in Pymol.

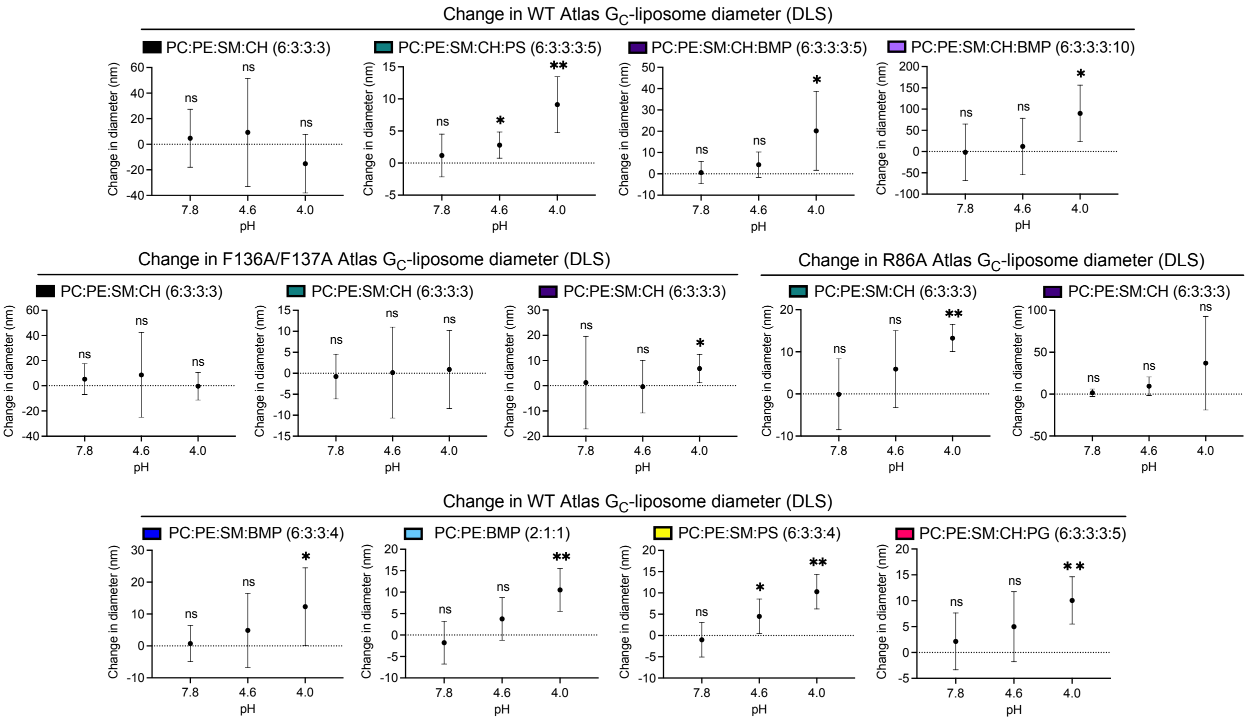

Fig. S7. Dynamic light scattering of Atlas G_C-liposome complexes.

Liposome diameter dynamic light scattering (DLS) data from **Fig. 4B** are shown as the mean change in liposome diameter upon binding Atlas G_C ectodomain (WT, F136A/F137A or R86A). Error bars show the standard error of the mean (s.e.m.) of three to seven replicates (see **dataset S1** for source data). Significance was determined by 2-way ANOVA analysis using Sidak's multiple comparisons test with a 95% confidence interval, in Prism 8 (GraphPad). **, $0.001 < p < 0.01$; *, $p < 0.05$; ns, not significant.

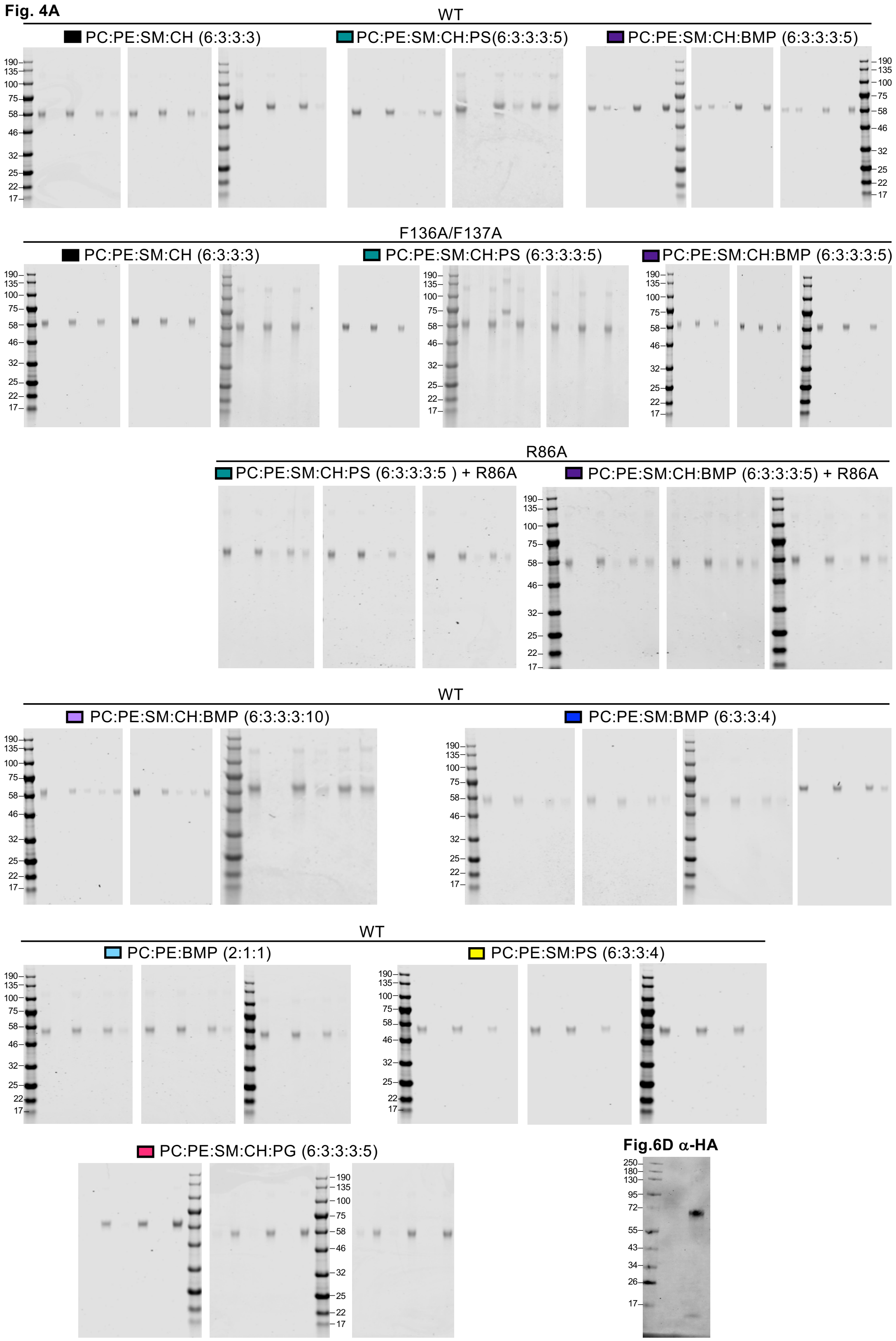

Fig. S8. Uncropped gels.

Gels shown for all liposome flotation replicates (**Fig. 4A**), along with immunoblot from **Fig. 6D**.

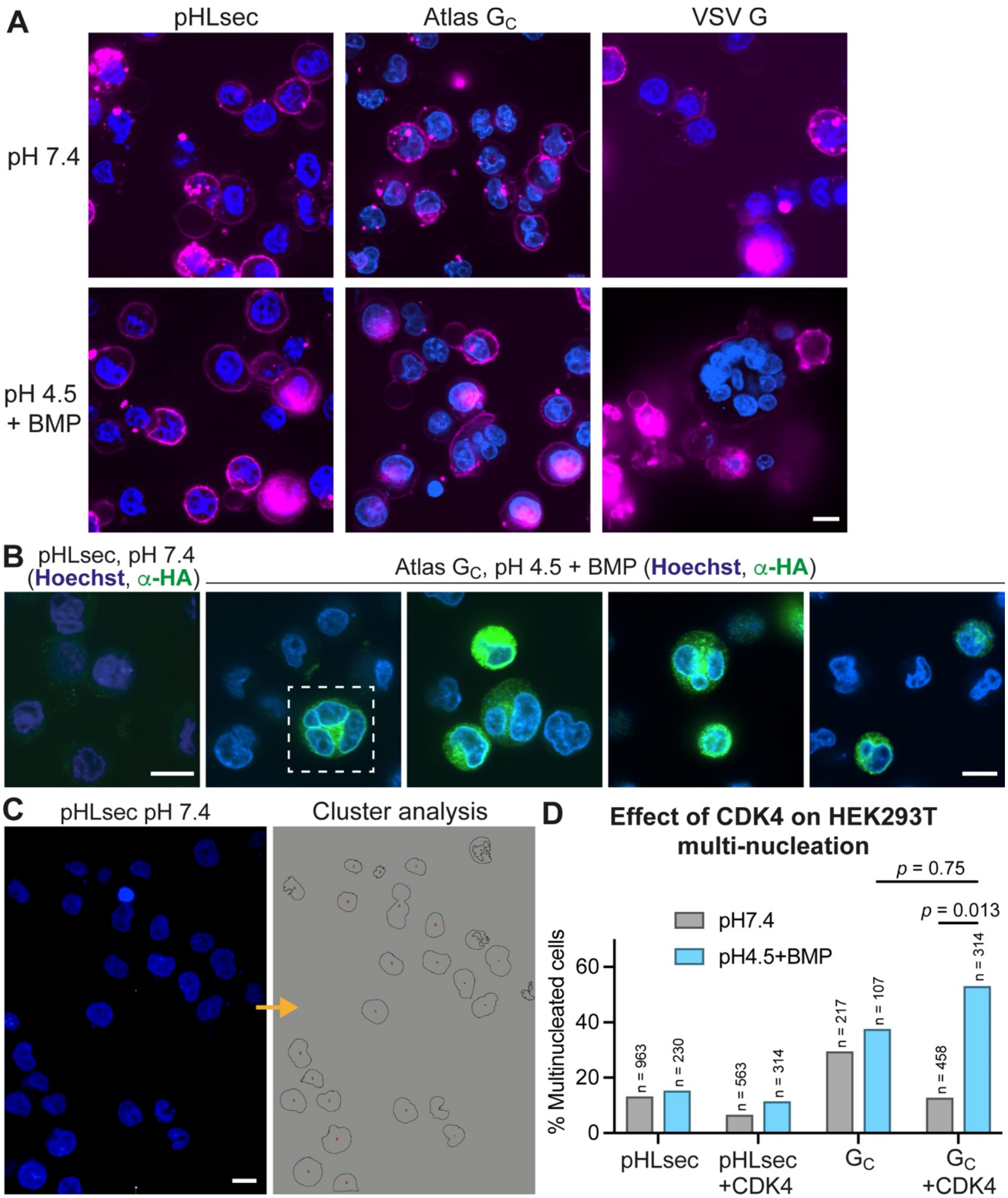

Fig. S9. Cell-cell fusion assay with CHO cells.

(**A**) Representative confocal micrographs of CHO cells transfected with plasmid encoding Atlas G$_C$, VSV G, or no protein (pHLsec empty vector). Cells were transferred to pH 4.5 medium containing BMP, or pH 7.4 medium without exogenous lipid. Blue, Hoechst 33342 nuclear stain; Magenta, CellBrite Red plasma membrane dye. (**B**) Permeabilized and fixed cells transfected with plasmid encoding G$_C$ stained with anti-HA antibody to detect the C-terminal HA tag on G$_C$. Blue, Hoechst 33342; green, anti-HA antibody. The boxed region is also shown in **Fig. 6B** and **Movie S1**. (**C**) Left, confocal micrograph of cells transfected with pHLsec plasmid stained with Hoechst 33342. Right, masks from cluster analysis of the Hoechst channel with Fiji (*83*). All scale bars are 10 µm. (**D**) Effect of cell cycle inhibitor CDK4 on Atlas G$_C$-induced cell multinucleation. Following treatment with BMP and pH 4.5, HEK293T cells were incubated in complete medium (pH 7.4) containing 10 µM CDK4 for 6 h before fixation. *n*, number of nuclei counted. See **dataset S2** for source data.

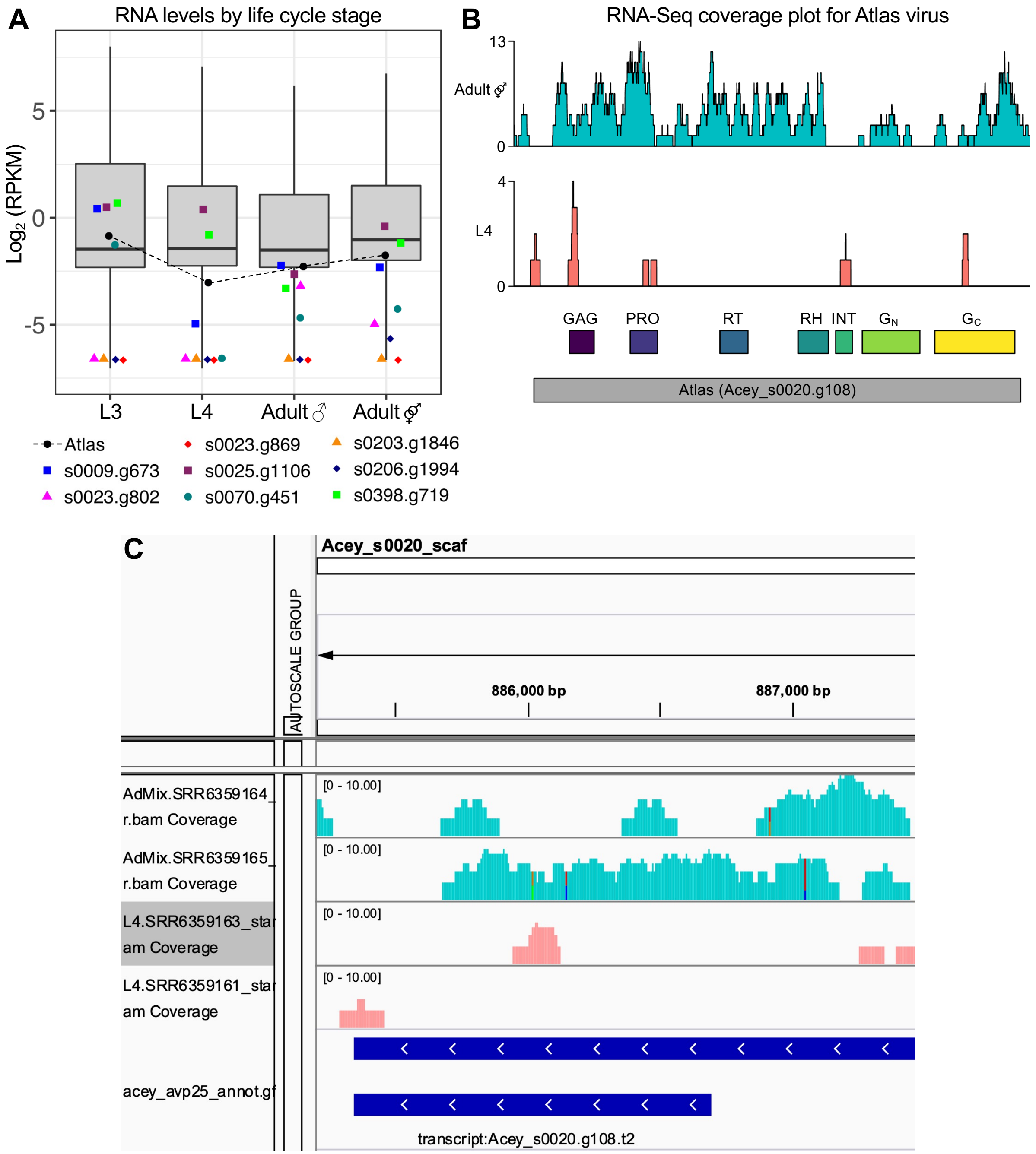

Fig. S10. RNA-Seq analysis of intact *A. ceylanicum* belpaovirus EVEs.

(**A**) Box plots of RNA-Seq Reads Per Kilobase of transcript per Million mapped reads (RPKM) for all annotated *A. ceylanicum* genes, at different developmental stages (L3 and L4 larva, adult male, adult mixed sex). RNA-Seq data source: (*69*). Lines in each box represent the median. Box boundaries represent the first and third quartiles. RPKM values are shown for nine intact belpaovirus EVEs including Atlas virus. Generated with ggplot2 (*84*) and karyoploteR (*85*). (**B**) Distribution of RNA-Seq reads over the Atlas virus coding sequence for the adult (mixed sex) and L4 developmental stages. (**C**) Integrative Genomics Viewer snapshot showing the distribution of raw RNA-Seq reads over the Atlas virus (*Acey_s0020.g108*) coding sequence in mixed sex adult worms and L4 larvae with individual unmerged BAM files.

	Atlas G_C
**Data Collection and Processing**	
Microscope	FEI Titan Krios
Voltage (kV)	300
Electron exposure (electrons Å^{-2})	46.12
Exposure per frame (electrons Å^{-2})	1.28
Defocus range (μm)	-1.3 to -3.5
Pixel size (Å)	1.047
N. initial particles	987,570
N. final particles	197,145
Map resolution (Å)	3.76
Map resolution range (Å)	3.50 - 5.20
FSC threshold for resolution limit	0.143
**Model refinement**	
Map sharpening B factor (Å^2)	-180
Symmetry imposed	C3
Mask correlation coefficient	0.78
**Model composition**	
N. non-hydrogen atoms	10,159
Protein residues (chains A, B, C)	1,320
**R.m.s. deviations**	
Bond lengths (Å)	0.005
Bond angles (˚)	0.694
Planarity (Å)	0.004
B*-factors/ADPs	
Minimum	35
Maximum	176
Mean	82
**Validation (Phenix 1.15)**	
MolProbity overall score	2.23
MolProbity all-atom clashscore	5.86
Rotamer outliers (%)	2.28
Ramachandran plot	
% Favored	86.2
% Allowed	13.8
% Outliers	0.0
PDB code	7A4A
EMDB code	EMD-11630
EMPIAR code	10266

Table S1. Cryo-EM data collection, structure determination, model building and refinement parameters and statistics.

ADPs, atomic displacement parameters.

Movie S1. Confocal Z-stack of the multinucleated cell shown in Fig. 6B (separate file).
CHO cell expressing Atlas G_C following treatment with pH 4.5 and BMP. Blue, Hoechst 33342; magenta, CellBrite Red; green, a-HA antibody for Atlas G_C detection. Scale bar, 10 μm.

Dataset S1. (Separate file)
Source data file for Fig. 4 – liposome binding assays.

Dataset S2. (Separate file)
Source data file for Fig. 6 and Fig. S9 – cell-cell fusion assays.

Dataset S3. (Separate file)
Source data file for Fig. S10 – original RNA-Seq annotation (GFF) file.

Dataset S4. (Separate file)
RNA-Seq analysis scripts and instructions (also available on Github [https://github.com/annaprotasio/Merchant_et_al_2020]).
